# Development of hybrid nanoparticles based on Zr(iv) and perylene-3,4,9,10-tetracarboxylic acid with visible-light photoredox activity

**DOI:** 10.1039/d5ra09148a

**Published:** 2026-02-02

**Authors:** R. Daniel Cacciari, Eduardo Gonik, Ana M. Beltrán, Martin D. Mizrahi, Sergio D. Ezquerra Riega, Hernán B. Rodríguez, Mónica C. Gonzalez

**Affiliations:** a Instituto de Investigaciones Fisicoquímicas Teóricas y Aplicadas (INIFTA), CCT-La Plata-CONICET, Universidad Nacional de La Plata Calle 113 y 64 La Plata Argentina gonzalez@inifta.unlp.edu.ar mcgonzalez.quim@gmail.com; b Departamento de Ingeniería y Ciencia de los Materiales y del Transporte, Escuela Politécnica Superior, Universidad de Sevilla 41011 Sevilla Spain; c CONICET-Universidad de Buenos Aires, Instituto de Química Física de los Materiales, Medio Ambiente y Energía (INQUIMAE) Ciudad Universitaria Pab. II C1428EHA Buenos Aires Argentina

## Abstract

Herein, we investigate perylene-3,4,9,10-tetracarboxylic dianhydride (PTCDA) as a linker in Zr-clusters. The photostable, 3D metal–organic nanomaterial obtained by a solvothermal synthesis procedure in the presence of formic acid as modulator, named ZIPER, shows strong absorption in the visible (400–560 nm) and an intense photoluminescence (PL) in the 600–700 nm range. PL quenching experiments strongly indicate that the ZIPER excited state (ZIPER*) behaves primarily as a strong oxidant and a mild reductant with redox couples *E*(ZIPER*/ZIPER˙^−^) = 1.8–1.2 V and *E*(ZIPER˙^+^/ZIPER*) = −0.44–−0.48 V (*vs.* NHE). Amine quenching of ZIPER* PL led to a strong reductant (ZIPER˙^−^) with *E*(ZIPER/ZIPER˙^−^) <−0.6 V *vs.* NHE. This reactivity was exploited to drive the reductive dehalogenation of model polychlorinated compounds, such as carbon tetrachloride and trichloroacetic acid, through visible-light photoredox catalysis in aqueous suspension. In contrast, under air-saturated conditions, the system predominantly produces substantial amounts of H_2_O_2_. A detailed analysis of the results suggests that photoexcitation of the organic linkers is followed by electron transfer to the Zr cluster. Charge-separated states are mainly stabilized in the presence of suitable electron donors or acceptors; otherwise, the system relaxes radiatively, emitting strong orange fluorescence.

## Abstract 2

Zr–perylene hybrid suspensions enable solar-driven photocatalytic redox reactions.

## Graphical abstract

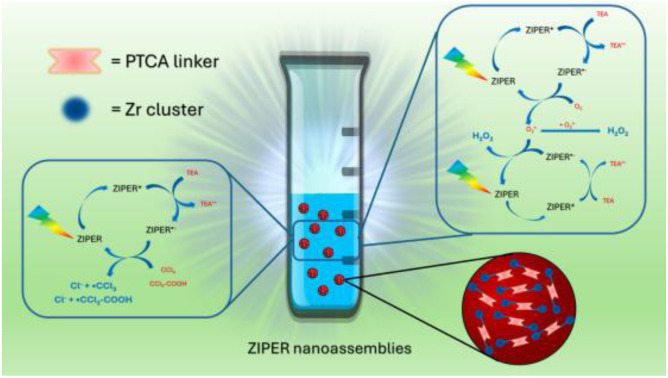

## Introduction

The presence of multiple interacting chromophores in a system leads to unexpected competitive processes that impact the material's light-induced phenomena,^1^ either by reducing its photoactivity or by generating new optical and electronic properties. A variety of excited-state dynamics have been reported due to the coherent mixing of states occurring upon chromophore aggregation. Among these, singlet fission and symmetry-breaking charge separation (SB-CS), are actively investigated for their use in optoelectronic devices, while excimer formation is often regarded as a trap.^2^ In particular, SB-CS involves the generation of charge carriers through electron transfer between two identical molecules after photoexcitation of one of them, as reported for cofacially-stacked perylene bisimide dimers^3^ and perylene in highly concentrated solutions.^4^ Thus, controlling interchromophoric interactions through proper arrangement of the molecular assemblies allows the evolution of unusual optoelectronic functionalities, offering unique photophysics and exciton dynamics.

Few studies in the literature examine the appearance of optoelectronic properties originating from interchromophoric interactions as a function of chromophores' spatial orientation. Such ordered chromophore systems can be obtained through supramolecular self-assembly, as seen in metal–organic frameworks (MOFs). MOF assemblies with chromophore linkers organized in a dense and highly periodic manner may exhibit emissive excited state features influenced by interchromophoric interactions in the ground state and through excited-state complex formation. The magnitude of these processes varies with linker orientation and proximity, as determined by the underlying framework. Moreover, such arrays of self-assembled chromophores also exhibit high absorptivity. In fact, data on a series of porphyrin and pyrene-based Zr(iv) MOFs^5^ describe the impact of topology-dependent interchromophoric interactions in the organic linkers on singlet-state emissive spectral evolution, optical bandgap, and excited state lifetimes.^6^

In addition, the interaction between ligands and clusters are reported to enhance organic materials photocatalytic performance allowing for ligand-to-cluster charge transfer (LCCT) processes responsible for the photocatalytic activity of MOFs.^7^ LCCT was reported for 5,10,15,20-tetra(4-carboxyphenyl)porphyrin-Zr (and Hf) metal organic materials under visible light irradiation.^8,9^ Moreover, it was demonstrated that LCCT lead to the reduction of Zr(iv) within Zr-oxo clusters to Zr(iii) in PCN-222, which further promoted hydrogen peroxide generation.^10^

Rylene dyes are emerging as promising building blocks to create π-functional materials as they exhibit excellent chemical and thermal stabilities, high electron affinities, remarkable electron-transporting properties, and strong reducing abilities in the excited state.^11^ In this work, we investigate perylene chromophores as linkers of Zr clusters, as they may potentially generate new pigments with optoelectronic properties that can tune exciton lifetime, and delocalization, and migration lengths, which are useful for technological applications. Perylene has been used as a chromophore for photo-driven reduction reactions due to its strong visible absorption (*ε*_440 nm_ = 38 500 M^−1^ cm^−1^) and strongly reducing excited state (−1.7 V *vs.* SCE).^1^ However, due to its singlet excited-state lifetime of ≈4 ns, efficient electron transfer to an acceptor requires a strong coupling between molecules. A multichromophoric assembly, with chromophores in proximity for interchromophore electronic coupling, may help avoid nonproductive competitive processes.^1,6,12,13^ Moreover, Zr(iv) metal ions where chosen as inorganic nodes due to their strong Zr–carboxylate bonds, which produce stable networks and possess an ‘inert’ atomic electronic configuration (*e.g.* diamagnetic), making them ideal for spectroscopic studies.^5,6,13^ To this end, perylene-3,4,9,10-tetracarboxylic acid (PTCA) was used as the organic linker of Zr-clusters. The hybrid material obtained through a solvothermal synthesis procedure in the presence of formic acid as a modulator was named ZIPER, reflecting its basic composition of Zr_6_-clusters and perylene organic linkers. The photochemistry and photoinduced electron transfer quenching properties of these assemblies were investigated in suspension.

A 3D metal organic material composed of Zr clusters and *N*,*N*′-di-(4-benzoic acid)-1,2,6,7-tetrachloroperylene-3,4,9,10-tetracarboxylic acid diimide ligand has been reported in the literature and used for stabilizing photoinduced radical anions in the solid phase.^14^

## Experimental section

All reactants and details of the standard equipment used (Fourier transformed infrared spectroscopy with total attenuated reflectance (FTIR-ATR), UV-Vis spectrophotometer, X-ray Diffraction (XRD), thermal gravimetric analysis (TGA), surface area determination (BET), and proton nuclear magnetic resonance (^1^H NMR)) are described in the SI (SI Reactants and Equipment, respectively).

**Scheme 1:**
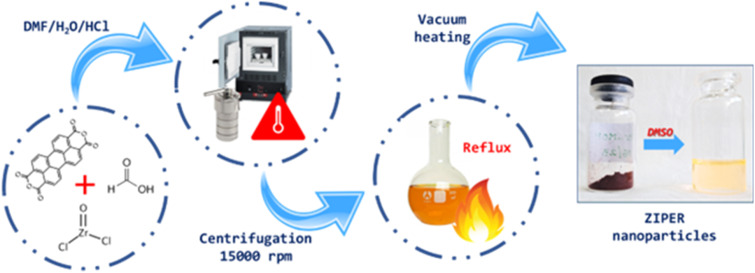
Synthesis pathways of ZIPER metal organic assemblies.

Perylene–Zr clusters assemblies were obtained by optimizing a solvothermal method reported for MOF-808 synthesis^15^ where perylene-3,4,9,10-tetracarboxylic acid (PTCA) was used as an organic linker. Perylene-3,4,9,10-tetracarboxylic dianhydride (PTCDA) was used as the precursor. Briefly, 0.1025 g PTCDA and 0.092 g ZrOCl_2_ were suspended in 10 mL of a 2.6 : 2.6 : 1 v/v mixture of DMF: H_2_O : 2 M HCl, also containing 0.1132 mL of formic acid used as a modulator. Only in the presence of water and acid, the reaction proceeds as expected. The mixture was sonicated for 15 min before filling a closed Teflon reactor which was further heated at 190 °C for 12 h in an oven. The obtained product mixture was centrifuged at 15 000 rpm for 15 min and the remaining solids were two times washed with DMF to eliminate undesired reactant residues. The solid product was then resuspended in 25 mL methanol and refluxed for 48 h to allow the solvent exchange in the nanoparticles' pores. Finally, activated metal organic assembly powder was obtained after drying the methanol solution in a vacuum oven for 5 h. The material thus obtained is named ZIPER. The synthesis procedure is illustrated in Scheme 1.

### Structural characterization

To understand the structure and composition, several techniques were used.

Transmission electron microscopy (TEM) observations were carried out on an FEI Talos F200S field-emission gun (FEG) microscope operated at 200 kV. Samples were prepared by depositing the powders onto copper support grids coated with an amorphous carbon film. Energy-dispersive X-ray spectroscopy (EDX) analyses were performed in scanning transmission electron microscopy (STEM) mode using a Thermo Fisher Talos F200X G2 instrument equipped with a Super-X EDX detection system consisting of four windowless silicon drift detectors (SDDs). Considering that a C film Cu grid was used and that EDX is not suitable for light element, the detection of C is qualitative. It must be emphasized that MOF structures are extremely sensitive to electron-beam irradiation, a limitation that may also affect the present material. Therefore, TEM and HAADF-STEM/EDX analyses should be interpreted with caution, as they were not conducted under ideal low-temperature and low-dose electron-beam conditions.^16^

Atomic Force Microscopy (AFM) images were obtained under air atmosphere using a MultiMode Scanning Probe Microscope (Veeco) equipped with a Nanoscope V controller (Veeco). All measurements were done, after fixation of a ZIPER suspension in tetrahydrofuran onto muscovite mica sheets, using probes doped with silicon nitride (RTESP, Veeco with tip nominal radius of 8–12 nm, 271–311 kHz, force constant 20–80 N m^−1^). Typical scan rates were 0.5 Hz.

The X-ray photoelectron spectroscopy (XPS) spectra were obtained under Ultra High Vacuum with a XR50 Specs GmbH spectrometer using Mg K(α) as the excitation source and a PHOIBOS 100 half sphere electron energy analyzer. A two-point calibration of the energy scale was performed using sputtered cleaned gold (Au 4f_7/2_, binding energy (BE) 84.00 eV) and copper (Cu 2p_3/2_, BE: 932.67 eV) samples. Internal calibration to correct for surface charging was performed with the C 1s peak at BE = 284.6 eV due to adventitious carbon. High resolution XPS spectra was taken to get a better insight into the chemical environment of the different atoms. A Shirley-type background from each spectrum was used to remove the effect of the extrinsic structure loss and the spectrum was resolved by Gaussian–Lorentzian fitting, keeping *χ*^2^ values to their minimum values. Typical FWHM were at 1.7 eV, except for the peaks attributed to satellites which were left free.

XAS (XANES + EXAFS) spectra were taken on solid samples using the in-house X-ray absorption spectrometer R-XAS Looper from Rigaku, in transmission mode at RT. Absorbents were prepared from fresh powder in pellets of 13 mm diameter and sealed with Kapton® tape (50 µm in thickness). The optimal amount of material for the measurements was determined using the XAFSMAS software.^17^ The radiation from the X-ray tube was directed through a 2 mm slit to a monochromator crystal Si (620) and then collimated by a second 10 × 0.2 mm rectangular slit. The intensities of incident and transmitted X-rays were measured using a Xe-filled proportional counter and a scintillation counter, respectively. XAS spectra were collected from 17 850 to 18 450 eV, with 1 eV steps, reduced to 0.5 eV in the XANES region (17 980–18040 eV). XANES spectra of reference compounds, Zr^0^ and ZrO_2_, were also recorded. The incident photon energy was calibrated using the first inflection point of the Zr K-edge (17 998 eV) from a reference foil of metallic Zr. For the ZIPER sample, ten spectra were acquired with exposure times of 1.5 hours each, later averaged. Background subtraction and edge-step normalization of the spectra were performed using the AUTOBK algorithm implemented in Athena, a component of the IFFEFIT package.^18^ The quantitative analysis of the EXAFS results was performed by modelling and fitting the isolated EXAFS oscillations. The EXAFS oscillations *χ*(*k*) were extracted from the experimental data with standard procedures using the Athena program. The *k*^2^ weighted *χ*(*k*) data, to enhance the oscillations at higher *k*, were Fourier transformed. The Fourier transformation was calculated using the Sine filtering function. EXAFS modelling was carried out using the LARCH software.^19^ Theoretical scattering path amplitudes and phase shifts for all paths used in the fits were calculated using the FEFF9 code.^20^ The *k*-range was set from 2.5 to 10.0 Å^−1^. The passive reduction factor *S*_0_^2^ value was restrained to 0.9. These values were obtained from the fitting standard foils of metallic Zr and constraining the coordination numbers to those corresponding to the structure.

### Physicochemical assays

All physicochemical and photophysical assays were performed using ZIPER dispersed in DMSO (unless otherwise specified) as a stable colloidal suspension of nanoparticles. Under the experimental conditions employed, the suspension is optically clear and shows no visible precipitation for at least two weeks, well beyond the time scale of the measurements.

Photoluminescence quantum yields (*Φ*) of ZIPER suspensions in DMSO at 25 °C were determined using acridine orange (AO) in dry ethanol as emission reference (*Φ*_em_ = 0.17 ± 0.01).^21^ The same excitation (*λ*_exc_ = 500 nm) and detection setup was used in all cases for samples and reference. Suspensions (solution) used were of <0.05 absorbance at the excitation wavelength to avoid inner-filter effects. The refractive index at 25 °C of reference and samples solvents, dry ethanol (*n* = 1.30) and DMSO (*n* = 1.43), respectively, were used for the corrections in quantum yield determinations. The temperature was controlled to ±0.1 °C with an F-3004 Peltier sample cooler controlled by a LFI-3751 temperature controller (wavelength electronics).

To correctly represent absorption and emission spectra together, the transition dipole moment (TDM) representation^22^ was considered, in which, the absorption spectrum was divided by the first power of the wavenumber and the emission by the third power. Moreover, for the estimation of the 0–0 transition energy and the Stokes shift, fluorescence and absorption spectra in the frequency scale were used. Considering that conversion of fluorescence from wavelength to energy requires further correction of the intensity to preserve spectral density, final fluorescence intensity *F*(*hv*) and absorbance *A*(*hv*) in the TDM representation corrections are *F*(*hv*) = *F*(*λ*) × *c* × *h*/(*hν*)^5^ and *A*(*hv*) = *A*(*λ*)/(*hv*), with “*c*” the velocity of light and “*h*” the Planck constant.

#### Quenching experiments

ZIPER suspensions in DMSO with 0.1 absorbance at 500 nm were bubbled with Ar for 20 minutes and titrated with different concentrations of quenchers. The suspensions were irradiated with light of 500 nm and ZIPER PL was detected between 505 and 780 nm. Triethylamine (TEA), triethanolamine (TEOA), methyl viologen (MV^2+^), and CuSO_4_ as a source of Cu^2+^ ions, were used as quenchers.

Methyl Viologen (MV^2+^) assays were performed with Ar-saturated ZIPER suspensions of 0.1 absorbance at 520 nm in the presence of 5 × 10^−5^ M MV^2+^ and 5 mM of either TEOA or TEA. The generation of the radical cation MV˙^+^ was monitored by its absorbance at 610 nm, where only the radical cation can absorb light.

Dehalogenation experiments were performed with trichloroacetic acid (TCA) and carbon tetrachloride (CTC) as proof molecules. To that purpose, 100 mL of an aqueous solution of either 2 mM TCA or 3 mM CTC, respectively, were mixed in a glass reactor with either 5 or 10 mg ZIPER (50 and 100 ppm final concentration) and TEA to a 50 mM final concentration. The reactor was purged with Ar gas for 20 min, sealed, and then irradiated with 520 nm light. Samples were taken at different irradiation times, centrifuged for ten minutes at 13 000 rpm, and 400 µL of the supernatant titrated with AgNO_3_ 0.01 M in the presence of K_2_CrO_4_ until the appearance of a red precipitate of Ag_2_CrO_4_.

Oxidation of 9,10-diphenylanthracene (DPA) upon 520 nm light irradition of a ZIPER suspension was followed by the decrease in absorbance of DPA at 410 nm. To that purpose, a 0.1 absorbance suspension of ZIPER in DMSO was put in a 1 cm optical path glass cuvette and bubbled with O_2_ gas before irradiation with 520 nm light for different periods of time. A long-pass cutoff filter (*λ* > 493 nm) was employed to ensure that DPA was not photolized during the experiment.

## Results and discussion

### Metal–organic assemblies size and morphology

**Fig. 1:**
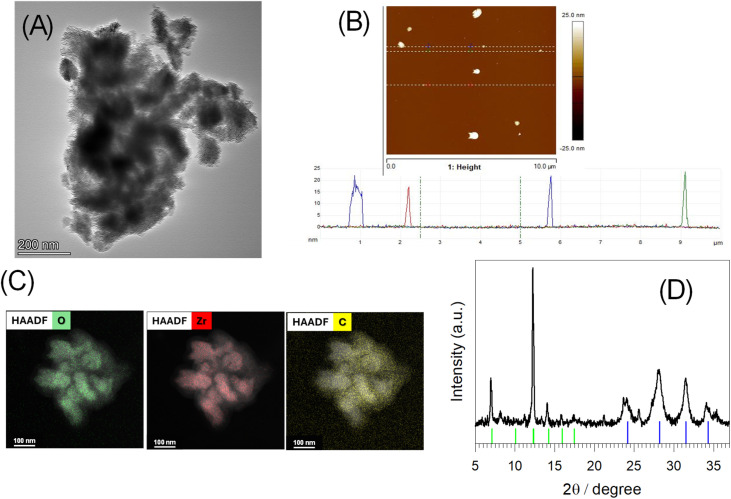
ZIPER (A) TEM images. (B) AFM and height profile in the direction of the white dashed lines of the main figure. (C) EDX elemental mapping images showing the distribution of O, Zr, and C elements in the particles HAADF. (D) Powder X-ray diffraction (PXRD, Cu Kα, *λ* = 0.15406 nm) of the samples. Black: experimental pattern; (—) body-centered cubic structure with inversion and threefold rotational symmetry model (*Im*3̄, *a* = 17.6 Å); (—) reference pattern of monoclinic ZrO_2_ (see text).

TEM images of ZIPER (Fig. 1A and SI Figure TEM) show irregular ellipsoidal-like nanoparticles of *ca.* 100 nm size with corrugated surface. Corresponding AFM images (see Fig. 1B and SI Figure AFM) depict particles width varying between 100 and 215 nm and heights varying from 7 to 23 nm, respectively, thus confirming oblate shaped nanoparticles. Differences in nanoparticles' width obtained from TEM and AFM may be assigned to AFM's lateral resolution which is affected by the shape of the probe.

High-angle annular dark-field (HAADF) imaging combined with EDX elemental mapping was used to reveal the distribution of Zr and O in the nanoparticles.^16^Fig. 1C show that both Zr and O signals extend across the entire particle, giving an overall homogeneous appearance, though with internal intensity variations. The two signals broadly overlap, indicating co-localization (see SI Figure SI Zr–O-HAADF overlay), with no evidence of large Zr-rich or O-rich segregates. Carbon appears more diffuse, with weaker intensity and higher background, as expected from its low atomic number and contribution from the amorphous C support. Nevertheless, C is still detected in the same regions as Zr and O. Within the detection limits and resolution of these EDX–HAADF maps, no phase separation is evident, although subtle segregation below the detection threshold cannot be excluded.

The XRD pattern of ZIPER (Fig. 1D) displays sharp low-angle Bragg reflections at 2*θ* = 7.1°, 12.3° (100% RI) and 14.2°, together with broader features at 2*θ* = 23.7–24.2°, 28.2°, 31.5° and 34.2°. The well-defined low-angle peaks are consistent with large *d*-spacings and long-range order and can be indexed by a body-centered cubic model (space group *Im*3̄) with cell constant *a* = 17.6 Å, giving calculated positions for (110), (211) and (220) at 7.1°, 12.3° and 14.2°, respectively, in excellent agreement with experiment. Moreover, this structural model satisfactorily reproduces all diffraction features observed in the 15° < 2*θ* < 30° range. It is well documented in the literature,^23^ that, planar tetratopic linkers coordinated to 12-connected Zr_6_(µ_3_-O)_4_(µ_3_-OH)_4_(COO)_12_ nodes tend to form 4,12-connected nets such as ftw/shp, even with rectangular linkers. In analogy with 3,3′,5,5′-biphenyltetracarboxylate (bptc) linker (aspect ratio ∼1.45), perylene tetracarboxylate (PTCA; aspect ratio ≈ 1.54) can still assemble with large Zr_6_ SBUs, albeit with possible orientational disorder. Based on these considerations, the idealized *Im*3̄ model (body-centered cubic structure with inversion and threefold rotational symmetry) reproduces the main low- and middle angle reflections (green bars in Fig. 1D). Attempts to attribute the low-angle region to crystalline organic PTCDA (α/β) domains did not provide satisfactory matches, supporting a MOF-like periodicity rather than simple molecular packing. Scherrer analysis (*K* = 0.9; Cu Kα, *λ* = 0.15406 nm) of the low-angle reflections performed without considering the instrumental correction, yields correlation lengths of *ca*. 48–50 nm, which should be regarded as lower estimates of the true crystallite size. The mismatch between particle size (*vide supra*) and domain length points to a sub-particle multidomain microstructure, consistent with the observed peak broadening.

In contrast, the high-angle region with peaks at 28.2°, 31.5° and 34.2° is assignable to tetragonal/monoclinic zirconia (see blue stick pattern^24^ in Fig. 1D). Scherrer sizes extracted from these ZrO_2_ peaks are ∼17–18 nm (no instrumental correction).

Nitrogen adsorption–desorption isotherms at 77 K (see SI Sorption Isotherms) yield a BET surface area of 119 ± 1 m^2^ g^−1^ and a mesopore size distribution centered in the ∼14–19 nm range. Given the crystallographic model and domain sizes, this porosity is best ascribed to interparticle voids and/or defect-generated mesopores, rather than to ideal framework pores.

**Fig. 2:**
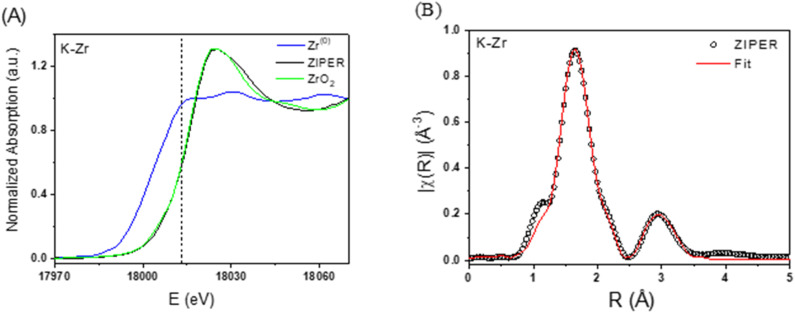
(A) XANES spectra measured in the Zr K-edge for ZIPER (black line) and the references Zr^0^ (blue line) and ZrO_2_ (green line). (B) Fourier Transformed EXAFS measured in the Zr K-edge for ZIPER (circles) and model fitting (red line).

The XANES spectrum of ZIPER was obtained to determine the average oxidation state of Zr atoms. Fig. 2A shows that the absorption edge energy of ZIPER aligns with that of reference ZrO_2_ (vertical dashed line), rather than metallic Zr, thus indicating that the average oxidation state of Zr in ZIPER is +4. Beyond the XANES region, the Extended X-ray Absorption Fine Structure (EXAFS) provides insights into the local structural environment around the absorbing atom. Refinement processes based on an initial structural model, through fitting techniques, can extract the average values of the coordination number (*N*), interatomic distances (*R*), and structural disorder (*σ*^2^). The Fourier transform (FT) of the EXAFS oscillation for ZIPER, shown in Fig. 2B (circles), reveals two main peaks corresponding to the first and second coordination spheres of Zr. A model assuming a single, uniform Zr–O distance in the first coordination shell fails to accurately fit the data. This suggests that either atoms of a different species are present in the first coordination sphere, or that oxygen atoms occupy distinct distances. Since the disorder parameter (*σ*^2^) for the first coordination sphere exceeds the expected values for a perfectly ordered structure with uniform distances, an improved model was required. A refined model accounts for two distinct Zr–oxygen distances within the first coordination sphere, specifically N_1_ oxygen atoms at one distance and N_2_ at a slightly different distance, along with Zr atoms as second neighbors. This approach successfully reproduced the experimental data (red curve in Fig. 2B). The fitting results indicate that ZIPER consists of Zr atoms coordinated by two oxygen atoms at an average distance of 2.08 Å and four additional oxygen atoms at 2.30 Å. The second coordination sphere comprises Zr atoms at an average distance of 3.45 Å, consistent with the expected structure of Zr_6_O_8_ clusters.^25^

The FT of the EXAFS oscillation for monoclinic ZrO_2_ was also obtained and compared to that of ZIPER. The mismatch between the EXAFS spectra of ZIPER and ZrO_2_ (see SI EXAFS spectra of ZrO_2_.) supports that the average Zr environment in ZIPER is Zr_6_O_8_-like, even in the presence of minor ZrO_2_ nanophases.

The combined EDX–HAADF, PXRD, and EXAFS analyses are consistent with ZIPER being MOF-like nanoparticles composed of Zr_6_-oxo clusters and perylene linkers, exhibiting local to medium-range order that can be described by a body-centered cubic structural model for the ordered fraction of the material, together with minor ZrO_2_ nanophases. High-angle PXRD indicates contributions attributable to ZrO_2_ with sizes of ∼17–18 nm, whereas XANES/EXAFS require Zr_6_O_8_-type environments and EDX–HAADF shows no particle-scale segregation. Overall, the data support ZrO_2_ occurring as minor, finely dispersed nanophases, likely intergrown with or surface-anchored to the same ZIPER particles.

### ZIPER composition

**Fig. 3:**
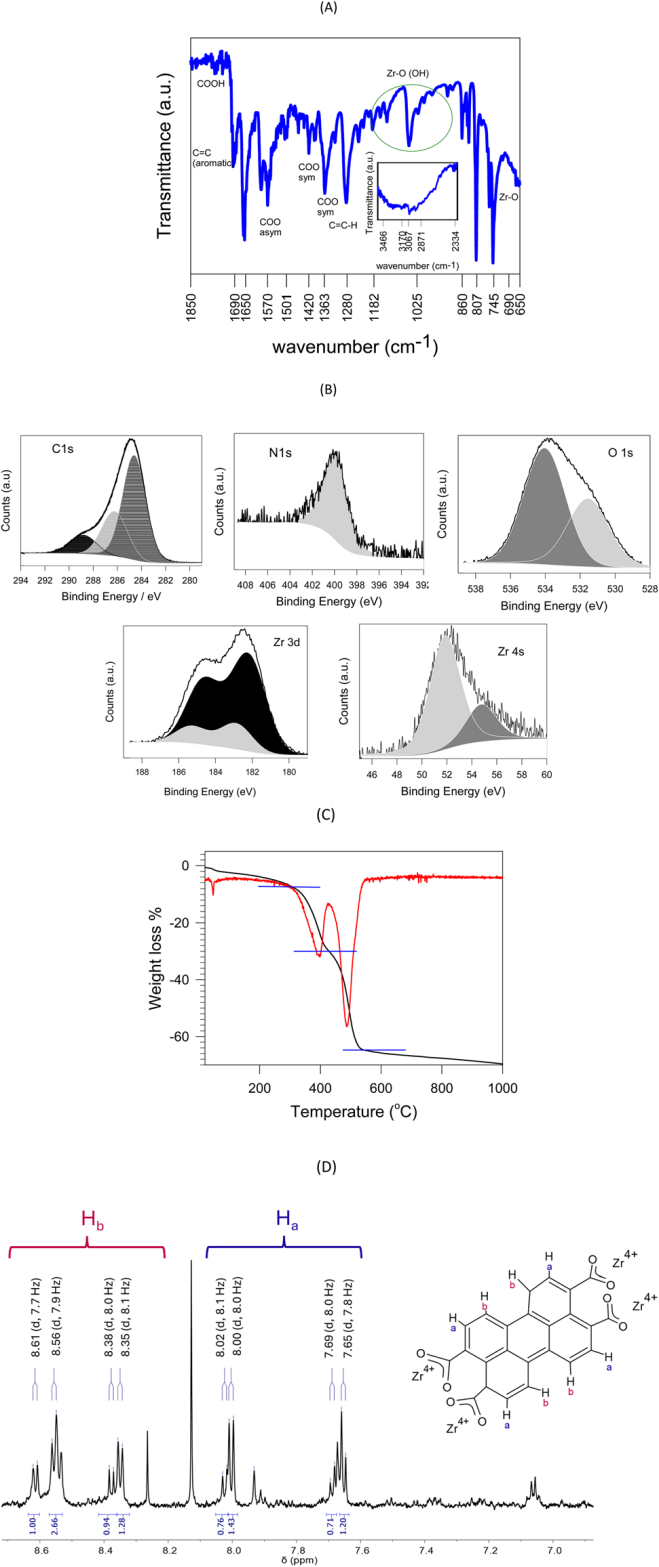
(A) FTIR spectrum of ZIPER. The inset displays the 3500–2300 cm^−1^ region. Tick labels show selected vibrational bands for clarity. (B) XPS spectra of ZIPER. (C) TGA (black lines) and first derivative (red lines). Blue lines delimit the mass loss of the linkers. (D) Aromatic region of the ^1^H-NMR spectrum (600 MHz) of ZIPER in DMSO-*d*_6_. “b” and “a” stand for bay and *ortho* hydrogen atoms, respectively.

Fourier transformed infrared spectrum of ZIPER dried samples show the presence of the aromatic carboxylic ligands and signals arising from the Zr cluster (see Fig. 3A; the complete FTIR spectrum is provided in the SI, Figure SI FTIR). The absence of the characteristic C–O–C bands of anhydrides appearing in 1732 and 1298 cm^−1^ and present in the PTCDA precursor, are a strong indication that PTCDA completely reacted to form the metal–organic assembly. Broad and intense bands in the 3600–2800 cm^−1^ spectral region, are assigned to the O–H bond stretching of water molecules.

Bands at 1650 and 1595 cm^−1^ originate in the strong C=C stretching of perylene aromatic rings,^26^ while that at 2950 cm^−1^ may be assigned to C–H vibrations of formate.^27^ The band at 1570 cm^−1^ characteristic of asymmetric COO stretching may show, both, the contribution of PTCA and formate carboxylates, while bands at 1365 and 1420 cm^−1^ originated in COO symmetric stretching mode may be due to formate carboxyl^27^ and to perylene carboxyl's in combination with other ring deformations, respectively. Unfortunately, the mixed nature of these vibrational modes is responsible for the splitting of the peaks making the assignment of the IR data challenging. In addition, the small bands in 1760 and 1735 cm^−1^ indicate the presence of free carboxylic acid groups, thus stressing that only a small fraction of PTCA carboxyl groups are not coordinating Zr atoms.^28^ Bands at 745 and 610 cm^−1^ are also characteristic of perylene vibration modes.

Bands at 1189 cm^−1^ may be assigned to Zr–OH and signals within 1055–1000 cm^−1^ are attributed to collective vibrations of the hydroxo/water ligands within the Zr_6_O_8_ node. The strong band at 745 cm^−1^ is assigned to the collective vibration involving *δ*(OH), *ν*(C–C) in perylene rings and *δ*(COO^−^) modes, while the broad band at 650 cm^−1^ is due to collective vibrations involving multiple Zr–O–C bonds of the Zr_6_O_8_ nodes within the assembly. The latter bands agree with the IR spectrum of MOF-808-P obtained from first principles calculations.^15^

XPS survey spectra obtained for ZIPER show that N, O, C and Zr are the main contributing atoms (see SI XPS Survey). Fig. 3B shows N 1s, C 1s, O 1s, Zr 3d, and Zr 4s peaks and the fitting considering the contributions of the atom's different environments. The XPS C 1s region displays the contribution of signals with BE 284.6, 286.3 and 288.8 eV characteristic of adventitious carbon, C–O and C–N, and C=O environments including carboxylate groups, respectively.^29^ The N 1s signal shows a main contribution with BE 399.6 eV characteristic of amide O=C–N environments. The XPS O 1s signal contributions at 531.5 (38%) and 534.0 (62%) eV may be assigned to Zr–O^30^ and organic O–C–O environments, respectively.^29^ The Zr 3d signal shows the characteristic Zr 3d_5/2_ – 3d_3/2_ splitting by 2.43 eV and two main contributions at Zr 3d_5/2_ BE of 182.2 and 182.9 eV, assigned in the literature to Zr–O and Zr(iv)–O–C environments, respectively.^29,31^ In addition we observed that Zr 4s signal also shows two main contributions, in line with Zr 3d observations.

The area ratio of N, O, and C to Zr peaks, each corrected by the atomic and instrument sensitivity factors, yield an average surface composition of Zr_1.0_C_14.8_N_0.25_O_6.4_. Considering that, any N atom present in the structure is due to adsorbed DMF (H_7_C_3_NO), that perylene tetracarboxylic acid formula is C_24_O_8_H_8_, and that the Zr node composition is Zr_6_O_4_(OH)_4_, a surface composition [Zr_6_O_4_(OH)_4_](DMF)_1.5_(perylene)_3.6_ is observed. In fact, an average number of 1.5 molecules of DMF are linked to each Zr node in ZIPER surface. The role of DMF is known to go beyond that of a solvent, as it may react as either an electrophilic or a nucleophilic agent capable of stabilizing MOFs structures.^32^ It should be recalled that, XPS results show the particle composition of an outer depth of *ca.* 8 nm.

TGA weight loss curves of ZIPER samples obtained under nitrogen atmosphere, see Fig. 3C, display two major losses in the 290–430 and 430–550 °C ranges. Considering that solvent release usually occurs below 280 °C and Zr transitions take place at temperatures >600 °C, the observed mass loss may be assigned to the elimination of the organic part of the composite. Since elimination of formic acid coordinated to Zr clusters has been observed *ca*. 150 °C,^33^ mass losses of 57.4% observed in the 300–550 °C range may be assigned to perylene molecules occupying positions of different lability, in line with ^1^H-NMR results showing different environments of the perylene molecules. At temperatures over 600 °C, Zr oxo-structures are known to predominantly produce tetragonal zirconia.^34^ Therefore, taking the molecular weights of ZrO_2_ and PTCA, an approximate Zr : PTCA molar ratio of 2.26 : 1 is estimated.

Considering that, a negligible ^1^H-NMR signal at 11.7 ppm (see Fig. 3D) corresponding to free acid hydrogens strongly supports the coordination of almost all PTCA carboxylates to the Zr node, in line with FTIR results, it may be assumed that most PTCA molecules are involved in a metal organic assembly. An ideal structure would involve Zr_6_(µ_3_-O)_4_(µ_3_-OH)_4_ node structures linked to three PTCA, as expected from the number of carboxyl groups in the molecule. Under such considerations, *ca.* 11% of Zr atoms are present as ZrO_2_.

Proton nuclear magnetic resonance (^1^H-NMR) spectrum of ZIPER in DMSO-*d*_6_ suspension is shown in Fig. 3D. *Ortho* and bay H proton resonances of perylene compounds appear as doublets located downfield, approximately between 7.0 ppm and 9.0 ppm, respectively. The presence of doublets with different shifts but with characteristic coupling constant *J* = 8.0 (±0.2) Hz,^35^ is indicative of the existence of perylene linkers in different magnetic environments. In fact, the existence of intense intermolecular interactions between perylene molecules may cause detectable changes in NMR chemical shifts, line shapes, and relaxation rates, as each perylene ring may affect the local magnetic field of adjacent molecules leading to ^1^H-NMR resonance shift and linewidth variations.^36^ The characteristic signals of DMF at 7.93, 2.88 and 2.72 ppm were also observed in agreement with previous results.^37^ The singlet at 8.12 ppm is assigned to formyl hydrogen (H–C=O) in formic acid^38^ used as modulator. The singlet at 8.28 ppm may be assigned to formyl hydrogen, in either formic acid or DMF, coordinated to Zr–O moieties in the metal–oxyhydroxide cluster.^39^

### Photoluminescence experiments

#### ZIPER photoluminescence general observations

**Fig. 4:**
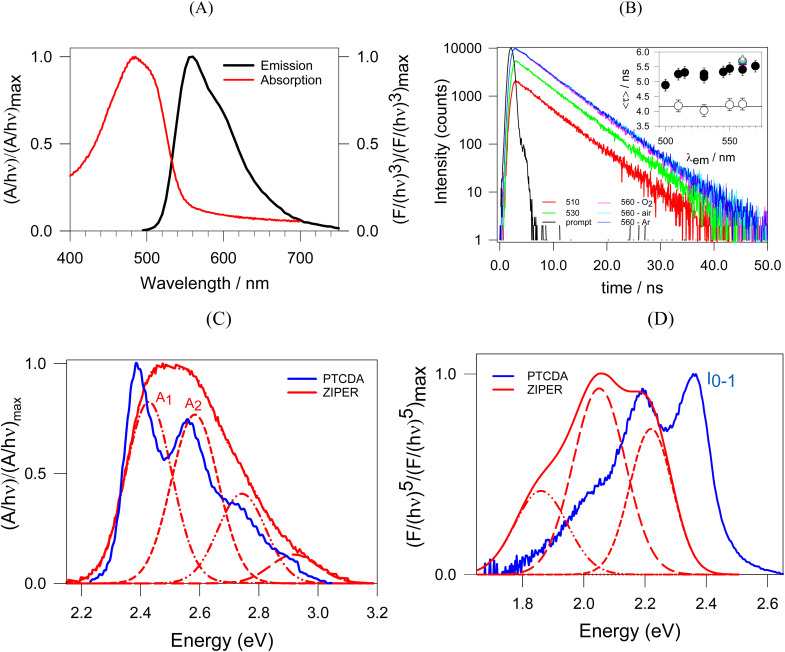
(A) ZIPER RT absorption spectrum (red lines) and emission at 490 nm excitation (black lines), normalized to their respective maxima. (B) RT emission decays obtained at different emission wavelengths upon 461 nm excitation of air saturated ZIPER suspensions in DMSO. Emission decays at 560 nm in oxygen and argon-saturated suspensions are also shown. Inset: mean half-life <*τ*> obtained from the fitting of the emission traces to a stretched exponential function *vs.* the emission wavelength for air-saturated (●) ZIPER and (○) PTCDA suspensions in DMSO at RT. *Δ* symbols (*λ*_em_ = 560 nm) stand for, from bottom to top, oxygen, air, and Ar-saturated suspensions, respectively. (C) PTCDA and ZIPER absorption spectra at RT. Red dashed lines stand for ZIPER deconvoluted peaks, with A_1_ and A_2_, the electronical absorption bands from the singlet ground state to the first excited singlet (S_0_→ S_1_) coupled to the first and second vibronic bands, respectively. Spectra were normalized to their maximum emission. (D) PTCDA and ZIPER emission spectra at RT. Red dashed lines stand for ZIPER deconvoluted peaks. Spectra were normalized to their maximum emission. *I*_0–0_ stands for PTCDA emission band from the 0 vibrational level of S_1_ to the 0 vibrational level of S_0_.

ZIPER room temperature (RT) absorption spectrum and emission spectrum upon 490 nm excitation are shown in Fig. 4A. Within the experimental error, the spectrum shape of ZIPER room temperature (RT) emission does not depend on the excitation wavelength, neither does the shape of the excitation spectrum depend on the emission wavelength used for its detection (see Figure SI Excitation and Emission Spectra). Excitation and absorption spectra are almost in coincidence, as shown in Figure SI Absorption Spectrum, thus suggesting that the contribution of scattered light is of little significance. Also, shape patterns of ZIPERs PL spectra are independent on ZIPER nanoparticles concentration in the range from 1.2 to 50 mg L^−1^, see Figure SI: effect of concentration on ZIPER PL. Therefore, under the experimental conditions used, it may be safely assumed that observed emission features originate within the main ZIPER nanoparticle structural network.

Emission decay traces were obtained in the nanosecond time range upon 461 nm excitation of ZIPER suspensions in DMSO. Suspensions were either bubbled for 20 minutes with Ar(g), air, or O_2_(g) and maintained in closed cells before excitation. Corresponding emission decay traces obtained at a given wavelength (see Fig. 4B), were fitted to a stretched-exponential function of the form 
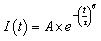
, which is suitable for describing a distribution of emitting states arising from perylene ligands with varying interactions. Notably, the observed heterogeneity (1/*β*) of the traces ranged from 0.98 to 1.02, strongly supporting a monoexponentially decay law. The characteristic decay time *τ* increases with emission wavelength, from 4.9 ns at 500 nm to 5.7 ns at 560 nm (see Fig. 4B inset). Comparable fluorescence decay times of 4.8 ns have been reported for the emission lifetime a tetraphenyl-pyrene-based Zr^IV^ MOF,^40^ in good agreement with our observations.

Considering that, ZIPER emission spectrum is invariant with respect to the excitation wavelength in the range from 350 to 560 nm, and that a small overlap between the absorption and emission spectra is observed between 500 and 550 nm, the progressive increase in decay time with emission wavelength within this region can be attributed to an additional dynamic non-radiative deactivation channel. In systems where chromophores are spatially confined within a rigid nanoscale framework, such behavior may be associated with non-radiative energy transfer processes. This analysis assumes that a single, relaxed emissive state is responsible for the emission across the spectral range and that the observed lifetime variation reflects the competition between radiative decay and an energy-transfer-mediated non-radiative channel, while the intrinsic radiative and non-radiative rates remain unchanged. Under these assumptions, the efficiency of non-radiative energy transfer can be estimated as,^41^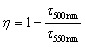 using the decay times observed at 500 nm and 550 nm, yielding an energy transfer efficiency *η* = 14%. Overall, the extracted efficiency should be regarded as an effective, ensemble-averaged parameter describing the relaxed emissive state, under the assumptions of averaged interchromophoric distances, orientational averaging, and nanosecond time resolution, while excluding possible ultrafast transient states formed at earlier times.

Altogether, the previous observations suggest that mainly one chromophore is responsible for RT ZIPER excitation-emission matrix. Both steady state emission spectra and fluorescence decay times, show no significant variations upon 20 minutes bubbling either with Ar(g) or O_2_(g) thus suggesting that the presence of dissolved molecular oxygen at varying concentrations had no significant effect on ZIPER PL. ZIPER quantum efficiency at RT is *Φ* = 0.73.

#### PTCDA photophysics

For further understanding the origin of ZIPER photoluminescence and considering that, PTCDA is the precursor of ZIPER organic linkers, PTCDA photophysical behavior will be discussed for comparison. The absorption spectrum of dilute solutions of PTCDA, in DMSO at RT (see Fig. 4C) depicts clear vibronic peaks at 512, 476, and 446 nm and a vibronic separation of 1452 cm^−1^ (0.18 (±0.01) eV) as shown by the separation between minima in the second derivative spectra (see Figure SI Vibronic Progressions (A)). Also, the observed Stokes Shift of *ca.* 0.03 eV is in line with absorbance and emission involving the same excited state. Observed absorption and emission features are in agreement with those reported for many π-conjugated, unaggregated chromophores, which dipole-allowed S_0_ → S_1_ electronic transitions are strongly coupled to a symmetric vinyl stretching mode nuclear coordinate with ∼0.15–0.18 eV energy.^42^ Also, argon-bubbled dilute PTCDA solutions exhibited a single fluorescence decay time of *τ* = 4.1 ns independent of emission wavelength (see Fig. 4B inset), consistent with reported values.^43^ PTCDA quantum efficiency at RT is *Φ* = 0.14.

#### Understanding ZIPER photophysics

Corresponding second derivatives of ZIPER RT absorption and emission spectra (see Figure SI Vibronic Progressions (B)) effectively depict minima separated by 0.18 (±0.05) eV. Also, deconvolution of ZIPER absorption and emission spectra assuming, as a first approximation Gaussian–Lorentzian line shapes for all transitions, yield vibronic bands (Fig. 4C and D) with separations within maxima in line with those observed by the second derivative analysis of the original spectra. These observations strongly indicate that ZIPER's electronic transitions are strongly coupled to the symmetric vinyl stretching mode nuclear coordinate consistent with the behaviour observed for perylene ligands.

The radiative rate constant *k*_rad_ can be calculated as *k*_rad_ = *Φ*/*τ*, where *Φ* = 0.73 and *τ* = 5.7 ns at 560 nm for ZIPER, and *Φ* = 0.14 and *τ* = 4.1 ns for PTCDA. This leads to *k*^ZIPER^_rad_ = 1.3 × 10^8^ s^−1^ and *k*^PTCDA^_rad_ = 3.4 × 10^7^ s^−1^, with *k*^ZIPER^_rad_/*k*^PTCDA^_rad_ = 3.8 at RT, strongly suggesting the higher emission capacity of ZIPER when compared to the free organic ligands.

Two main photophysical processes can be thought of to explain ZIPER's PL: excimer-like photophysics and Ligand to Cluster Charge Transfer (LCCT), in line with those reported for MOFs consisting of Zr-oxo clusters and either 2,6-naphthalene dicarboxylate^44^ and 5,10,15,20-tetra(4-carboxyphenyl) porphyrins^7^ as organic linkers.

Excimer like photophysics involves absorption and emissive features driven by the interactions of nearby ligands, which are strongly dependent on the orientation and proximity of the organic ligands within the underlying ZIPER framework. ZIPER visible absorption spectra depict band separations of *ca*. 0.18 eV, in line with those of PTCDA, though bands are broader, and blue shifted by 0.05 eV compared to the PTCDA ligand in solution (see Fig. 4C). Such signatures are frequently observed in perylene aggregates in a predominantly H arrangement.^45^ Within the Kasha/Frenkel model, the two main signatures identifying H-type aggregates when comparing with the monomeric form are, a blue-shift of the main absorbance peak and the attenuation of the fluorescence component, which adopts a vibronic progression without the *I*_0–0_ peak.^42^ In fact, absorption bands from the singlet ground state to the first excited singlet (S_0_→ S_1_) coupled to the first vibronic band A_1_ is observed at 2.43 eV. This peak, *I*_0–0_, is absent in the emission spectra. While the absence of *I*_0–0_ emission may account for the large Stokes shift observed, it cannot explain the significantly enhanced RT quantum efficiency of ZIPER with respect to that of PTCDA. Therefore, any aggregate contribution to the emissive excited states at RT may be discarded.

After light absorption by the photosensitive interacting perylene ligands, the presence of Zr-clusters in ZIPER's structure could influence charge transfer events leading to ligand to cluster charge transfer (LCCT) processes. To obtain evidence of a photoinduced LCCT event, photoexcited ZIPER exchange of electrons with electron donors and acceptors is further investigated.

### Electron transfer photoluminescence quenching

Electron transfer to or from an excited-state species with the formation of a highly reactive radical pair is of importance for future uses of ZIPER as organic photocatalyzer.

#### PL quenching by electron donors-reductive quenching

Fluorescence quenching of ZIPER by the aliphatic amines triethanolamine (TEOA) and triethylamine (TEA), well-known electron donors with *E*(TEAO˙^+^/TEAO) = 1.20 V and *E*(TEA˙^+^/TEA) = 0.83–0.88 V *vs.* NHE.^46,47^ was studied under steady-state and time-resolved conditions. Both amines efficiently quenched ZIPER emission according to the following sequence, with *k*_q_ the quenching constant.

R1 ZIPER + *hν* → ZIPER*

R2 ZIPER* → ZIPER + *hν*′

R3
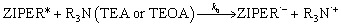


**Fig. 5:**
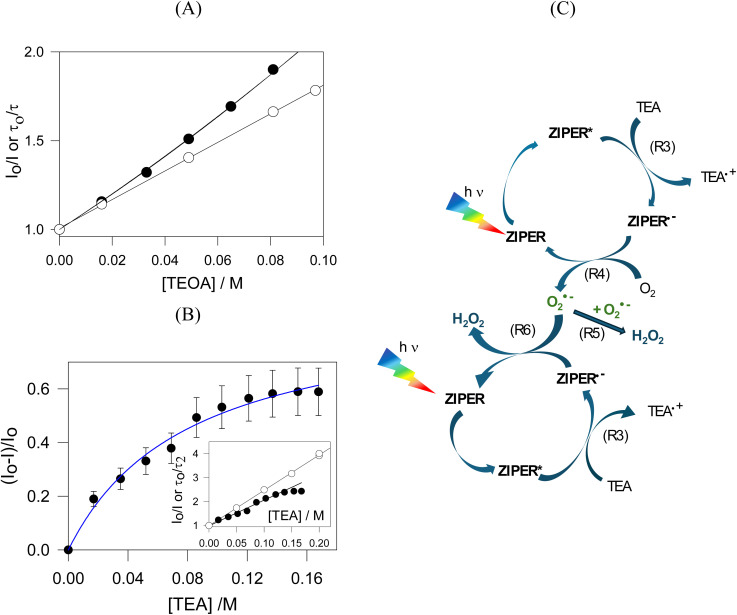
Quenching experiments by electron donors and corresponding redox cycles (A) (○) plot of *τ*/*τ*_0_*vs.* [TEOA] and fitting to the Stern Volmer eqn (1) (*r*^2^ = 0.999); (●) plot of the ratio between the PL intensity of ZIPER in the absence and presence of TEOA (*I*_0_/*I*) *vs.* [TEOA] and fitting to the effective volume model, eqn (2) (*r*^2^ = 0.996). (B) Fitting of the quenching data of ZIPER's PL by TEA in DMSO suspensions to the Lehrer equation (*r*^2^ = 0.986). Inset: (○) plot of *τ*_2_/*τ*_0_*vs.* [TEA] and fitting to the Stern–Volmer equation eqn (1) (*r*^2^ = 0.999). (●) Plot of the ratio between the PL intensity of ZIPER in the absence and presence of TEA (*I*_0_/*I*) *vs.* [TEA]. (C) Proposed redox cycle for the formation of H_2_O_2_ by ZIPER reductive performance in air-saturated suspension in the presence of 10 mM TEA.

**Scheme 2:**
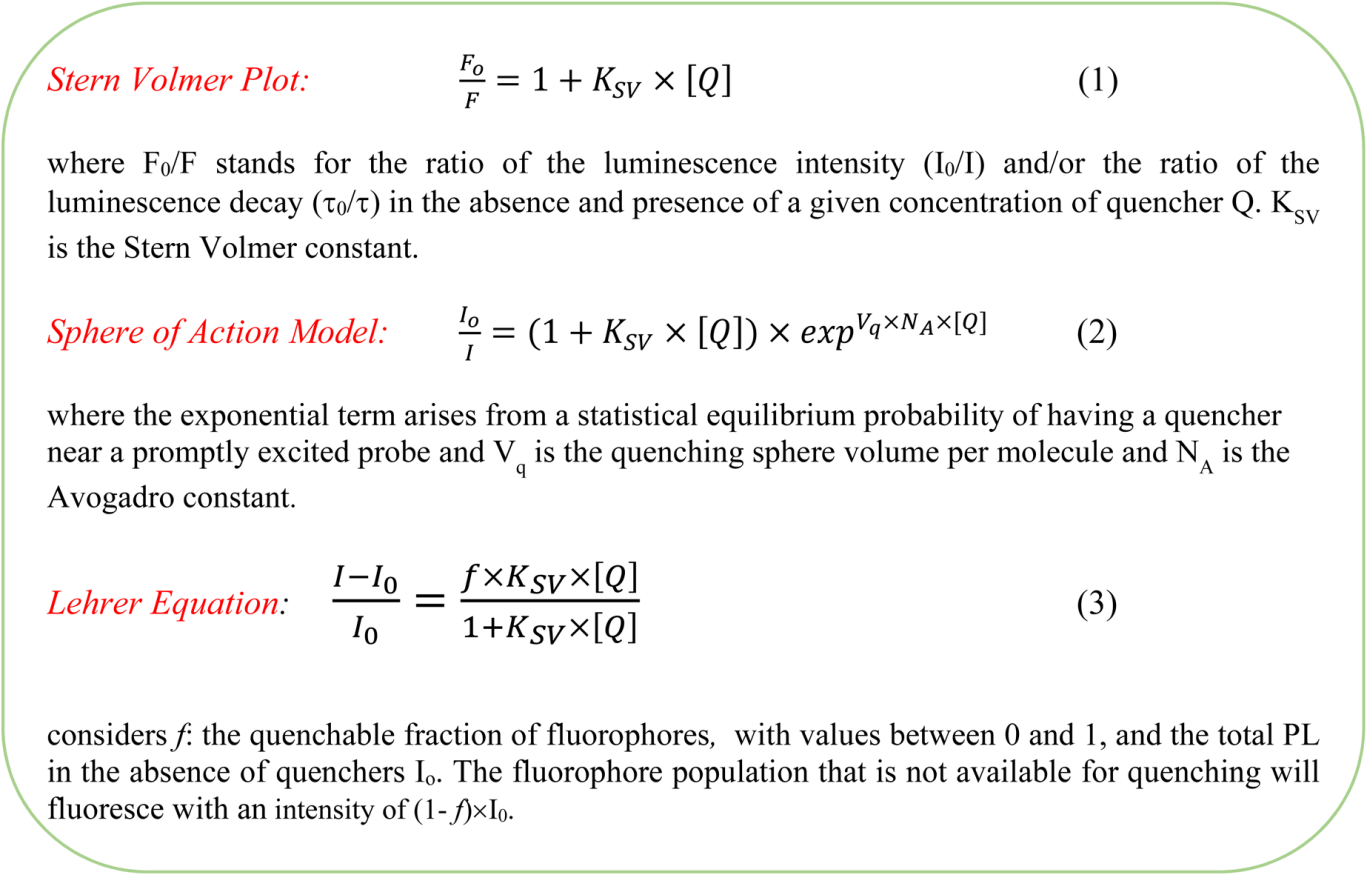
Fluorescence quenching equations according to the different models used.

Both dynamic and steady-state Stern–Volmer analyses for TEOA gave *K*_SV_ = (8 ± 0.1) M^−1^ (*r*^2^ > 0.99, see Fig. 5A), consistent with a combined dynamic and static sphere-of-action quenching model (see Scheme 2) with *V*_q_= (2.8 ± 0.1) nm^3^.^48^ In heterogeneous solid–liquid systems as in ZIPER suspensions, *V*_q_ should be interpreted as an effective interfacial proximity region around accessible emissive sites that can be quenched without requiring diffusive encounters during the excited-state lifetime. The estimated effective distance (*d*_q_ ≈ 0.9 nm) is consistent with the length of the perylene core (*ca* 1.4 × 0.42 nm^2^).^49^

For TEA, Stern–Volmer plots obtained from steady state quenching experiments depict a downward/negative deviation, as shown in Fig. 5B inset. Negative deviations may be associated with emitters which cannot be reached by quencher molecules and data should be treated following the Lehrer equation, see Scheme 2.^50^ Lehrer analysis indicated a fraction (*f* ≈ 0.9) of accessible chromophores and *K*_SV_ = (12 ± 2) M^−1^. Stern–Volmer plots of the dynamic component yield *K*_SV_ = (14.8 ± 0.3) M^−1^. Further details of these analyses are given in the SI Reductive Quenching.

Considering *K*_SV_ = *k*_q_ × *τ*_o_ and a mean decay time obtained in the emission wavelength range *τ*_o_ = 5.3 ns, quenching rate constants *k*_q_ = 1.5 × 10^9^ and 2.8 × 10^9^ M^−1^s^−1^ are obtained for TEOA and TEA, respectively. These values are close to diffusion-controlled limits and in line with that reported for TEA quenching of perylene fluorescence in acetonitrile (*k*_q_ = 3 × 10^9^ M^−1^s^−1^).^51^

The differing accessibility of the quenchers to ZIPER emissive sites likely originates from polarity effects rather than steric hindrance, since TEA is smaller than TEOA, which bears bulky hydroxyl terminal groups. Given the markedly different dielectric constants of TEA (*ε* = 2.42) and TEOA (*ε* = 37.7), it seems reasonable to assume that *ca.* 9% of ZIPER chromophores are located within hydrophilic domains where TEA interaction is hindered.

Photolysis experiments at 520 nm of air-saturated ZIPER (5 mg L^−1^) DMSO suspensions in the presence of 10 mM of TEA or TEOA, showed the formation of 440 ± 10 and 420 ± 10 µM h^−1^ of hydrogen peroxide, respectively, while [H_2_O_2_] <10 µM h^−1^ was detected in the absence of donors. Even after 1 h of Ar-saturation of ZIPER suspensions in the presence of TEA, *ca*. 300 µM h^−1^ of H_2_O_2_ were generated, strongly evidencing the presence of residual O_2_ after intense purging.

Luminescence quenching by one donor molecule leads to the formation of a reduced ZIPER radical anion (ZIPER˙^−^), defining a photocatalytic process governed by sequential single-electron transfer steps. The ZIPER˙^−^ radical anion can subsequently transfer an electron to O_2_ to form superoxide (O_2_˙^−^), reaction (R4) in Fig. 5C. Further disproportionation of superoxide may result in H_2_O_2_ formation, reaction (R5). Additional reduction of O_2_˙^−^ by a second ZIPER˙^−^, reaction (R6), may also occur. While a direct two-electron reduction of O_2_ to H_2_O_2_ under the applied conditions cannot be strictly excluded, in Zr-MOF systems such pathways have been primarily associated with the presence of organic redox mediators^52^ (*e.g.*, quinone-type units) or specific framework designs that enable charge accumulation and suppress H_2_O_2_ decomposition.^53^ In the absence of these features, oxygen reduction is more commonly discussed in terms of sequential single-electron transfer processes.

Definitive discrimination between sequential and concerted oxygen reduction pathways would require dedicated mechanistic studies beyond the scope of the present work.

**Fig. 6:**
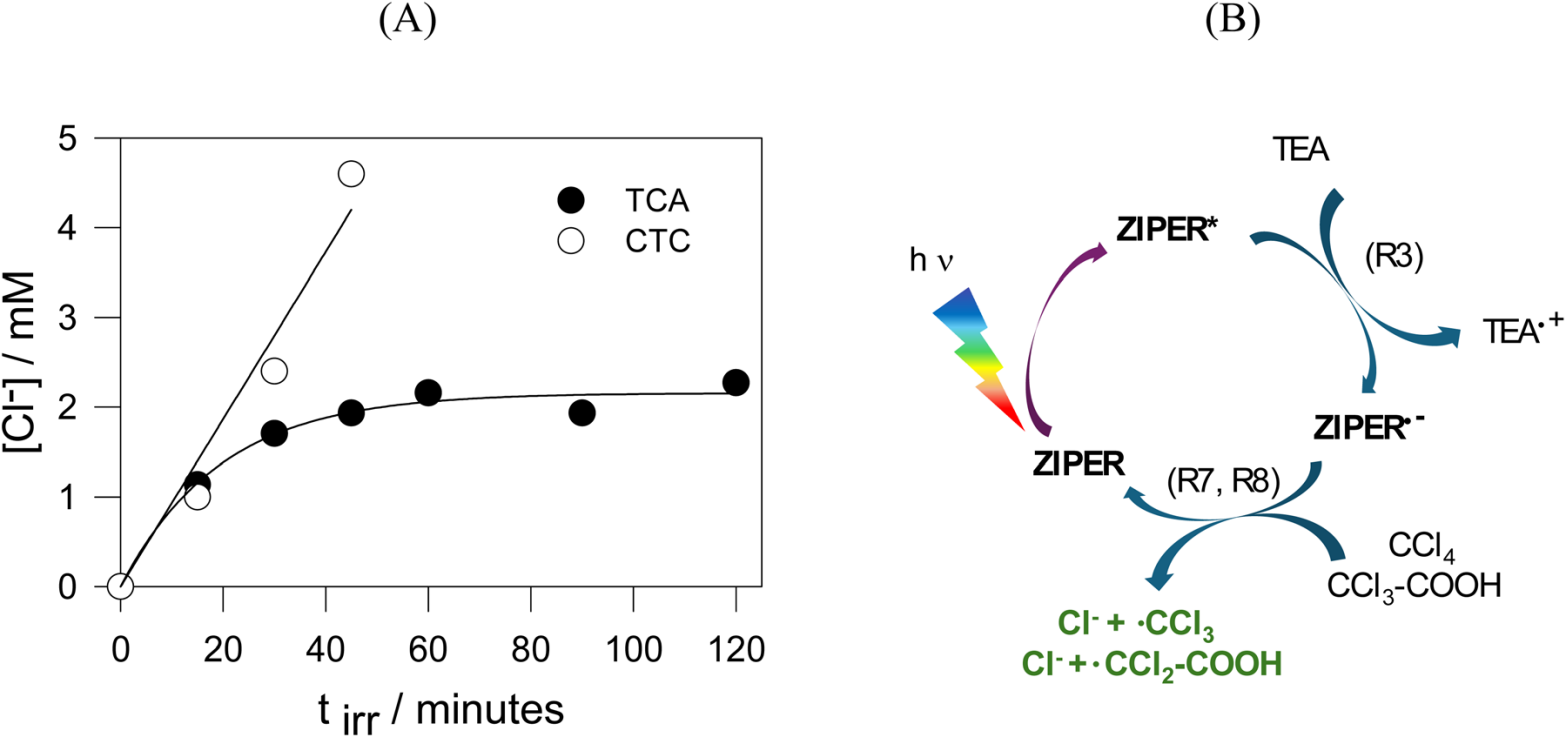
Proofs of ZIPER* oxidation capacity and reductive redox cycle. (A) Dechlorination of 3 mM CTC (○) and 2 mM CTA (●) observed upon 520 nm light irradiation experiments of deaerated aqueous suspensions containing 100 ppM ZIPER and 50 mM TEA. (B) Proposed redox cycle for ZIPER reductive performance in deaerated suspensions in the presence of TEA.

To further investigate the reductive performance of ZIPER/TEA under the action of visible light, the recalcitrant organic pollutants carbon tetrachloride (CTC) and trichloroacetic acid (TCA) were selected as target molecules capable of undergoing reductive dechlorination^54^ with reduction potentials *E*(CCl_4_/Cl^−^ + ˙CCl_3_) = −0.36–−0.48 V and *E*(CCl_3_COOH/Cl^−^ + ˙CCl_2_COOH) = −0.6 V *vs.* NHE, respectively.^55^ Therefore, irradiation experiments of ZIPER/TEA suspensions with 520 nm light were performed in the presence of 3 mM CTC and 2 mM TCA and formation of chloride anions was detected by the Mohr method. Chloride release was used here as a direct probe of C–Cl bond cleavage, while the fate of carbon-centered intermediates, which is known to be strongly donor- and medium-dependent, was not addressed.^56^Fig. 6A shows the formation of chloride anions as a funtion of irradiation time. Blank experiments in the absence of TEA do not lead to the formation of Cl^−^ anions. Consequently, the radical anion ZIPER˙^−^ is expected to be the main species reducing CTC and TCA, as shown in the proposed redox cycle in Fig. 6B, reactions (R7) and (R8).

On thermodynamic bases, for reactions (R7) and (R8) to occur, the condition *E*(ZIPER/ZIPER˙^−^) < *E*(CCl_4_/Cl^−^ + ˙CCl_3_), *E*(CCl_3_COOH/Cl^−^ + ˙CCl_2_COOH) should apply. Considering the one electron reduction potentials of CTC and TCA, *E*(ZIPER/ZIPER˙^−^) <−0.6 V. Taking *E*_0–0_ = 2.42 eV and considering that *E*(ZIPER*/ZIPER˙^−^) = *E*(ZIPER/ZIPER˙^−^) + *E*_0–0_,^57^ an upper limit value *E*(ZIPER*/ZIPER˙^−^) < 1.8 V *vs.* NHE is estimated. On the other hand, a lower limit value for this potential may be obtained from the thermodynamic condition *E*(TEA˙^+^/TEA), *E*(TEOA˙^+^/TEOA) < *E*(ZIPER*/ZIPER˙^−^) for the occurrence of reaction (R3). The fact that 1.20 V < *E*(ZIPER*/ZIPER˙^−^) < 1.8 V *vs.* NHE strongly supports that ZIPER excited state is an oxidant capable of stablishing reductive redox cycles.^58^ On this estimation, ZIPER* cannot oxidize H_2_O to HO˙ *E*(HO˙/H_2_O) = 2.73 V *vs.* NHE.

As additional evidence of the oxidative capacity of ZIPER*, 9,10-diphenylanthracene (DPA, *E*(DPAQ˙^+^/DPA) = 0.934 V *vs.* NHE) was employed as a redox probe. Photoluminescence measurements showed efficient quenching of ZIPER emission upon DPA addition, accompanied by measurable DPA depletion (see SI Reductive Quenching), indicating an oxidative quenching pathway. Because DPA is also a well-known singlet oxygen (^1^O_2_) scavenger, time-resolved laser experiments were performed on oxygen-bubbled ZIPER suspensions monitoring the characteristic ^1^O_2_ phosphorescence at 1270 nm. The absence of a detectable ^1^O_2_ phosphorescence signal, within the detection limit of the time-resolved near-infrared measurements (low nanomolar ^1^O_2_ concentration range), indicates that a photosensitized singlet-oxygen mechanism does not play a significant role in the observed photocatalytic behavior of ZIPER, consistent with direct electron-transfer oxidation of DPA by excited ZIPER*.

#### PL quenching by electron acceptors – oxidative quenching

Electron transfer between the excited state of ZIPER and an external electron acceptor was also investigated. Methyl viologen (MV^2+^, 1,1′-dimethyl-4,4′-bipyridilium dication), a well-known electron acceptor^59^ (*E*(MV^2+^/MV^+^) = −0.44 V *vs.* NHE) was employed to evaluate the formation of the oxidized radical species ZIPER˙^+^, according to reaction (R9).R4
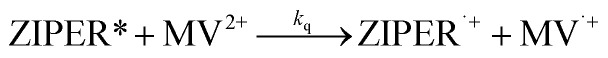


**Fig. 7:**
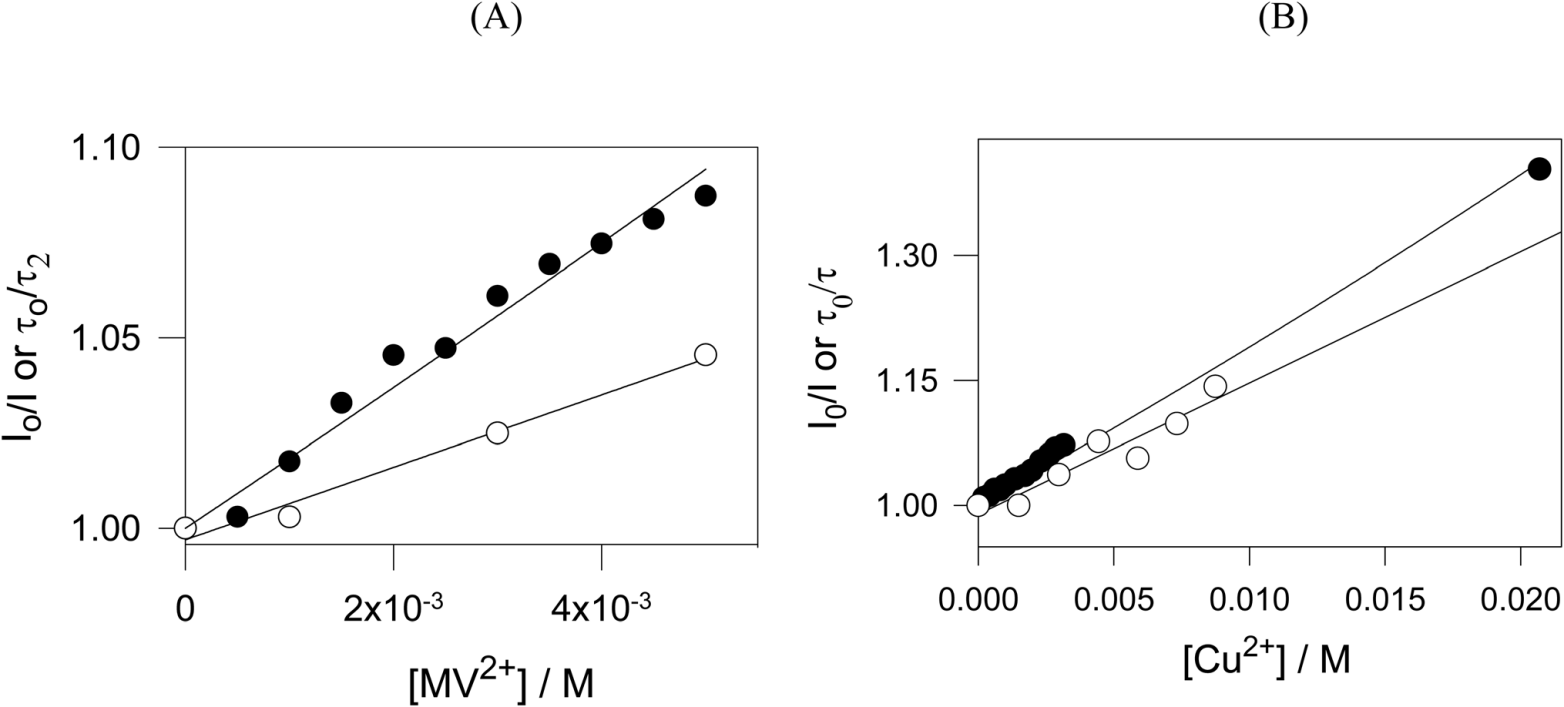
Quenching experiments by electron acceptors. (A) MV^2+^ experiments (○) plots of *τ*/*τ*_0_*vs.* [MV^2+^] and fitting to the Stern Volmer eqn (1) (*r*^2^ = 0.982); (●) plots of (*I*_0_/*I*) *vs.* [MV^2+^] and fitting to the effective volume model, eqn (2) (*r*^2^ = 0.974). (B) Cu^2+^ experiments. (○) Plots of *τ*/*τ*_0_*vs.* [Cu^2+^] and fitting to the Stern Volmer eqn (1) (*r*^2^ = 0.981); (●) plots of (*I*_0_/*I*) *vs.* [Cu^2+^] and fitting to the effective volume model, eqn (2) (*r*^2^ = 0.992).

Time resolved Stern–Volmer plots in Ar-saturated suspensions, (*τ*_o_/*τ*) *vs.* [MV^2+^], yield a linear dependence with *K*_SV_= (9.5 ± 0.1) M^−1^, see Fig. 7A, confirming a dynamic quenching with a diffusion-controlled quenching rate constant *k*_q_ = 1.8 × 10^9^ M^−1^ s^−1^, of the order of those observed for TEOA and TEA. Corresponding steady state data, (*I*_0_/*I*) *vs.* [MV^2+^], exhibited higher quenching ratios, indicative of a concurrent static contribution. Fitting to the sphere-of-action model equation, eqn (2) afforded a sphere of action volume *V*_q_ = (14.4 ± 0.8) nm^3^ corresponding to an effective distance *d*_q_ = 1.5 nm, comparable to the perylene core dimension.

Considering that, ZIPER* can reduce MV^2+^ but not CTC nor TCA, then, on thermodynamic bases −0.48V ≤ *E*(ZIPER˙^+^/ZIPER*)≤ −0.44 V, strongly indicating that ZIPER excited state may be a moderate reductant.

Quenching of ZIPER PL by Cu^2+^ ions was investigated under Ar-saturated conditions. Time resolved Stern–Volmer plots (see open symbols in Fig. 7B) displayed a linear dependence with *K*_SV_= (14 ± 1) M^−1^. Corresponding plots of (*I*_0_/m) *vs.* [Cu^2+^] show only slightly higher quenching rates than those observed for dynamic quenching, thus indicating a predominantly dynamic quenching process and a small contribution of static quenching.

Copper(ii) ions, being hard Lewis acids, are known to rapidly coordinate to carboxylate oxygen atoms of Zr-MOFs frameworks,^60,61^ typically leading to non-emissive Cu-bound sites. Such coordination would lead to irreversible, static quenching, which may account for the small static contribution observed. However, the predominance of a dynamic quenching component indicates the occurrence of electronic interactions between ZIPER's excited states and Cu^2+^ ions. In fact, considering a fluorescence decay time *τ* = 5.3 ns, a diffusion-controlled quenching rate constant *k*_q_ = 2.6 × 10^9^ M^−1^s^−1^ is estimated, comparable to that obtained for TEA quenching. Considering the redox potential E_cu^2+^/cu^+^_ = 0.153 V *vs.* NHE, these results strongly support an electron-transfer pathway involving reduction of Cu^2+^ by ZIPER*.

Altogether, quenching experiments strongly indicate that ZIPER* can donate and accept electrons depending on the nature of the quencher and it behaves primarily as a strong oxidant and a mild reductant with redox couples *E*(ZIPER*/ZIPER˙^−^) = 1.8–1.2 V and *E*(ZIPER˙^+^/ZIPER*) = −0.44–−0.48 V *vs.* NHE. Thus, strongly supporting that LCCT processes might contribute to ZIPER's RT emission.

## Discussion

Interchromophoric interactions between perylene linkers may, in principle, give rise to two distinct excited-state pathways: ligand–ligand excimer states or symmetry-breaking charge transfer (SB-CT), as reported for well-defined cofacial perylene dimers and highly concentrated molecular assemblies.^62,63^ As discussed earlier, excimer-like photophysics arising from a predominantly H-type π–π coupling between neighboring chromophores is typically manifested by broad, red-shifted emission bands, reduced photoluminescence quantum yields, and shortened or multiexponential excited-state lifetimes. In contrast, SB-CT involves ultrafast electron transfer between equivalent chromophores, leading to charge-separated states that are essentially non-emissive due to their low oscillator strength and dominant non-radiative decay. Neither of these characteristic signatures are observed for ZIPER. Accordingly, the high photoluminescence quantum yield, monoexponential nanosecond lifetimes, and excitation-independent emission profiles rule out excimer dominated relaxation and SB-CT as dominant excited-state pathways.

The experimental observations strongly support the following conclusions. (i) The absorption process in the 400–560 nm range arises from interacting perylene linkers assembled in ZIPER structure, resulting from the arrangement of PTCA linkers and Zr-oxo clusters. (ii) The observed PL originates after the relaxation of the ligands excited state to a ligand-to-cluster charge transfer (LCCT) state. Such LCCT states are built upon the displacement of a partial electronic density of the electron rich ligand to an unoccupied molecular orbital within the metal cluster.^64^ The singlet nature of the emission indicates that LCCT states possess singlet multiplicity, consistent with the absence of phosphorescence. (iii) Only in the presence of appropriate electron donors or acceptors, LCCT states evolve into long-lived charge separated states (CSS) while in their absence, radiative decay predominates. Similar LCCT-driven CSS formation has been reported for Zr-2,6-naphthalene dicarboxylate MOFs,^44^ particularly at defective sites. In fact, the inherent rigidity of the perylene tetracarboxylate linker in ZIPER may promote structural defects that facilitate CSS generation.

**Scheme 3:**
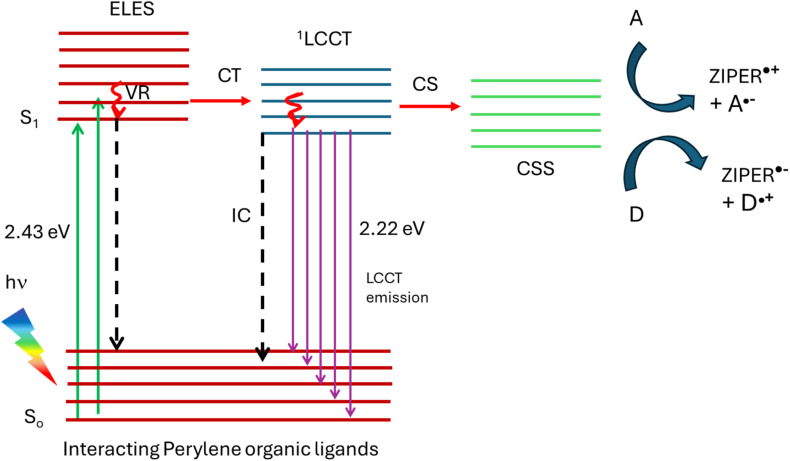
Diagram of possible processes taking place after ZIPER excitation with visible light at RT. S_0_ and S_1_: singlet ground and excited states, respectively; VR: vibrational relaxation; IC: internal conversion; CT: charge transfer, CS: charge separation; A: electron acceptor; D: electron donor.

Scheme 3 depicts a possible photoluminescence process after ZIPER photoexcitation in the visible range at RT. The initial event of light absorption by interacting perylene organic linkers leads to excimer-like excited states, ELES. However, conversion to a ^1^LCCT state of lower energy, might occur. Formation of charged separated states (CSS) occurs mainly in the presence of electron donors and acceptors. Otherwise, LCCT states decay mainly radiatively. In fact, ligand to metal charge transfer involving metals with low electronic density in d orbitals like Zr, are known to decay radiatively, like Zr(iv) complexes with π-donating ligands.^65,66^ The high luminescence quantum yield is consistent with a difficult non-radiative deactivation of the excited states due to the rigidity of the molecular structures. The formation of a photoinduced CSS, generating electrons and holes in ZIPER experiments using electron acceptors and donors, opens the way to their use as photocatalysts in diverse reactions.

Despite processes proposed in Scheme 3 may explain the observed results, further experiments, like time resolved transient absorbance in the fs and ns time domain and computational studies are necessary for getting insight in understanding ZIPER photophysics.

Fluorescence quenching experiments can reveal whether certain chromophores are inaccessible to quenchers, indicating that the quencher molecules are unable to diffuse close enough to the chromophores positions. This is, indeed, the case of TEA, which can only access a fraction *f* ≈ 0.9 of the emitting chromophores for quenching. However, TEA inability was suggested to be rather related to the hydrophilic nature of the surface, thus pointing to the heterogeneous nature of the particles surface. Other bigger quenchers as MV^2+^, Cu^2+^ and DPA did not depict quenching limitations due to their access inside pores, thus indicating that quenching reactions seem to take place on the particles surface, without the electron donors and acceptors getting inside the structures. Particle structural heterogeneity, as depicted by NMR, might favor ligand interactions capable of driving the transfer of the exciton to the surface within each nanoparticle, in line with literature reports indicating that strong short contacts across different directions can significantly enhance exciton diffusion.^67^

It should be recalled that, given the wide bandgap of ZrO_2_,^68^ minor zirconia nanophases present in ZIPER are not expected to contribute directly to visible-light photocatalysis; however, they may still play an indirect role by facilitating interfacial charge separation and suppressing charge recombination within the hybrid particles.^69^

## Conclusion

A photostable, partially ordered nanocrystalline PTCA-based 3D metal–organic assembly (ZIPER) was obtained by a one-pot synthesis procedure using a low cost, commercially available organic precursor, and an earth-abundant metal. Visible light irradiation of ZIPER suspensions in the presence of electron-donor amines enables efficient photoredox activity, as demonstrated by the reductive transformation of representative halogenated substrates and the formation of significant amounts of H_2_O_2_. Due to the repetitive arrangement of Zr-oxo clusters and perylene units within the ZIPER nanoparticles, the material behaves as an array of self-assembled molecular photoredox catalysts. These features make ZIPER a promising platform for solar-driven photocatalytic redox reactions.

Future studies will examine how solvent polarity, perylene substitution, and the nature of the electron donors and acceptors affect the photoredox activity of ZIPER.

## Conflicts of interest

The authors declare no conflicts of interest.

## Abbreviations

CTC: Carbon tetrachloride
DPA: 9,10-Diphenylanthracene
PL: Photoluminescence
TCA: Trichloroacetic acid
TEA: Triethylamine
TEOA: Triethanolamine
ZIPER: Zirconium-perylene metal organic assembly
PTCA: Perylene-3,4,9,10-tetracarboxylic acid
PTCDA: Perylene-3,4,9,10-tetracarboxylic acid diamide

## Supplementary Material

RA-016-D5RA09148A-s001

## Data Availability

The data supporting this article have been included as part of the supplementary information (SI). Supplementary information: list of reactants, equipment, photophysical experiments, TEM images, AFM, XRD diffractograms, EXAFS spectra of ZIPER and ZrO_2_, XPS survey, sorption isotherms, FTIR, excitation and emission spectra, absorption spectrum, effect of concentration on ZIPER PL, vibronic progressions, Tauc plot, methyl viologen quenching, hydrogen peroxide determination, detection of MV^+^ after photoinduced electron transfer. See DOI: https://doi.org/10.1039/d5ra09148a.
