# Supplementary material for: Development of hybrid nanoparticles based on Zr(iv) and perylene-3,4,9,10-tetracarboxylic acid with visible-light photoredox activity
Source: RSC Adv. 2026 Feb 2;16(7):6689–705. doi: 10.1039/d5ra09148a (PMC12862888; doi:10.1039/d5ra09148a)
Supplement: RA-016-D5RA09148A-s001 [file RA-016-D5RA09148A-s001.pdf]

Supplementary Information (SI) for RSC Advances.
This journal is © The Royal Society of Chemistry 2026

Supporting Information

Development of Hybrid Nanoparticles Based on Zr(IV) and Perylene-3,4,9,10-Tetracarboxylic Acid with Visible-Light Photoredox Activity.

*R. Daniel Cacciari[1], Eduardo Gonik[1], Ana M. Beltrán[2], Martin D. Mizrahi[1], Sergio D. Ezquerra Riega[3], Hernán B. Rodríguez[3] and Mónica C. Gonzalez[2]**

[1] Instituto de Investigaciones Fisicoquímicas Teóricas y Aplicadas (INIFTA), CCT-La Plata-CONICET, Universidad Nacional de La Plata, Calle 113 y 64, La Plata, Argentina.

[2] Departamento de Ingeniería y Ciencia de los Materiales y del Transporte, Escuela Politécnica Superior, Universidad de Sevilla, 41011 Sevilla, España

[3] Universidad de Buenos Aires, Instituto de Química Física de los Materiales, Medio Ambiente y Energía (INQUIMAE, UBA/CONICET), Ciudad Universitaria Pab. II, C1428EHA Buenos Aires, Argentina.

*Corresponding author E-mail: gonzalez@inifta.unlp.edu.ar; mcgonzalez.quim@gmail.com

Content

Reactants 3
Equipment 4
Infrared spectroscopy and X-ray diffraction 4
Reflectance measurements and Tauc analysis 4
Thermal Gravimetric Analysis 4
Adsorption-desorption isotherms – Surface Area determination 5
Nuclear Magnetic Resonance 5
Photophysical experiments 5
Absorption Measurements 5
Steady-state Measurements 5
Time-resolved Measurements 6
Figure S.I. TEM 7
Figure S.I. AFM 8
Figure SI Zr-O-HAADF Overlay 9
Figure S.I. DRX 10
Figure S.I. FTIR 11
Figure S.I. XPS Survey 12
Figure S.I. EXAFS spectra of ZIPER and ZrO_2 13
Figure S.I. Sorption isotherms 14
Figure S.I. Excitation and emission spectra 15
Figure S.I. Absorption spectrum 16
Figure S.I. Effect of concentration on ZIPER PL 17
Figure S.I. Vibronic progressions 17
Figure S.I. Tauc plot 18
Reductive Quenching 19
Methyl Viologen quenching. 22
Hydrogen peroxide determination 23
Detection of $MV^{\cdot+}$ after photoinduced electron transfer. 24

Reactants

N,N-dimethylformamide (DMF), ethanol 99.5%, triethanolamine (TEOA) 99%, carbon tetrachloride (CTC) were obtained from Cicarelli. Dimethyl sulfoxide (DMSO) and triethylamine (TEA) were purchased from Sintorgan. Zirconyl chloride octahydrate 98% ($ZrOCl_2$), perylene-3,4,9,10-tetracarboxylic anhydride (PTCDA), methyl viologen dichloride hydrate 98% (MV^{2+}), acridine orange 99% (AO) and trichloro acetic acid 95% (TCA) were purchased from Sigma-Aldrich chemical company. 9,10-diphenylanthracene 98% (DPA) was purchased from Fluka. $CuSO_4$ was from Biopack. All reagents were of analytical grade and used as received without further purification. Deionized water (>18 MΩcm, < 20 ppb organic carbon) was obtained using a Millipore system. Argon and oxygen gas were both from La Oxígena S.A., Argentina.

Equipment

Infrared spectroscopy and X-ray diffraction

IR spectrum of ZIPER was obtained with a Bruker EQUINOX 25 spectrometer using KBr disks as holders. Spectra were taken in the 4000-400 cm^{-1} range with 1 cm^{-1} resolution. Attenuated Total Reflection Infrared (ATR-IR) spectroscopy was performed with an Agilent Cary 630 equipment with an ATR single bounce accessory and a diamond crystal with an incident beam angle of 45°.

The crystalline phase content of the samples was analyzed by X-ray diffraction (XRD) using a Panalytical Empyrean X-ray diffractometer with the Cu anode (Cu-Kα radiation = 0.15406 nm) equipped with a $PIXcel^{3D}$ field detector. The radiation source generator operated at an accelerating voltage of 40 kV and an applied current of 44 mA. The detection range was set from 4 to 40° under θ/2θ configuration.

Reflectance measurements and Tauc analysis

The diffuse reflectance spectrum for ZIPER was recorded with a double-beam PGI-T90+ UV-vis spectrophotometer equipped with a $BaSO_4$ integrating sphere in a solid sample holder. To apply the Tauc method for energy gap calculations, the absorption of light (A) by the solid was obtained from the Kubelka-Munk equation, using the diffuse reflectance data.

Thermal Gravimetric Analysis

Thermal gravimetric analysis (TGA) of the powders was performed using a DTA-TA Thermo Plus EVO 2 instrument. The program used a N_2 environment (60 mL/min) and involved a 1-min initial temperature of 50 °C, followed by 10 °C/min ramp up to 1000 °C and maintaining at this temperature for 10 min.

Adsorption-desorption isotherms – Surface Area determination

Nitrogen gas-volumetric adsorption−desorption isotherms for specific surface area determinations were performed in a Micromeritics equipment ASAP 2020 V1.02 E. Before each measurement, the samples were dried overnight at 100 °C at a residual pressure of about 10^{-2} mbar to guarantee a good cleaning of the sample surface.

Nuclear Magnetic Resonance

Proton nuclear magnetic resonance (^{1}H-NMR) spectrum of ZIPER samples were recorded in DMSO-*d6* solution on NMR Bruker AVANCE III 600 MHz (14.1 T) Spectrometer at room temperature (25 ± 2 °C). Chemical shifts (δ) are reported in parts per million (ppm), referenced to the solvent peak, DMSO-*d6* defined at δ = 2.50 ppm. Spin multiplicities as singlet (s), doublet (d), doble doublet (dd), triplet (t), and multiplet (m). Coupling constants (J) are given in Hz.

Photophysical experiments

Absorption Measurements

All absorption spectra recorded throughout this study were measured in a double-beam Shimadzu UV-3600 spectrophotometer in a 1 cm quartz cuvette at a scan rate of 300 nm/min and 1 nm bandpass, employing the correspondent solvent as blank.

Steady-state Measurements

Steady-state PL measurements were performed with a Jobin-Yvon Spex Fluorolog FL3-11 spectrometer. The temperature was controlled to ± 0.1 ºC. Excitation-emission matrices (EEM) of nanoparticles suspensions in DMSO were obtained in the wavelength range 300-500 nm and 320-700 nm for excitation and emission, respectively. In all cases, sample attenuance was adjusted to 0.05 at 350 nm, to avoid inner-filter effects. All spectra were corrected for the detection system responsivity with wavelength and the emission spectrum of the excitation source, while Raman scattering signals were subtracted by

using the corresponding emission spectrum of the solvent.

Time-resolved Measurements

Time-resolved photoluminescence measurements were carried out in the same Jobin-Yvon Spex Fluorolog FL3-11 spectrometer with the aid of a time-correlated single photon counting (TCSPC) module. The pulsed excitation was done using a 461 nm LED (FWHM ~ 1.21 ns) operating at 1 MHz repetition. The instrumental response function (IRF) was obtained at the corresponding excitation wavelength using a Ludox scattering suspension.

Figure S.I. TEM

TEM images.

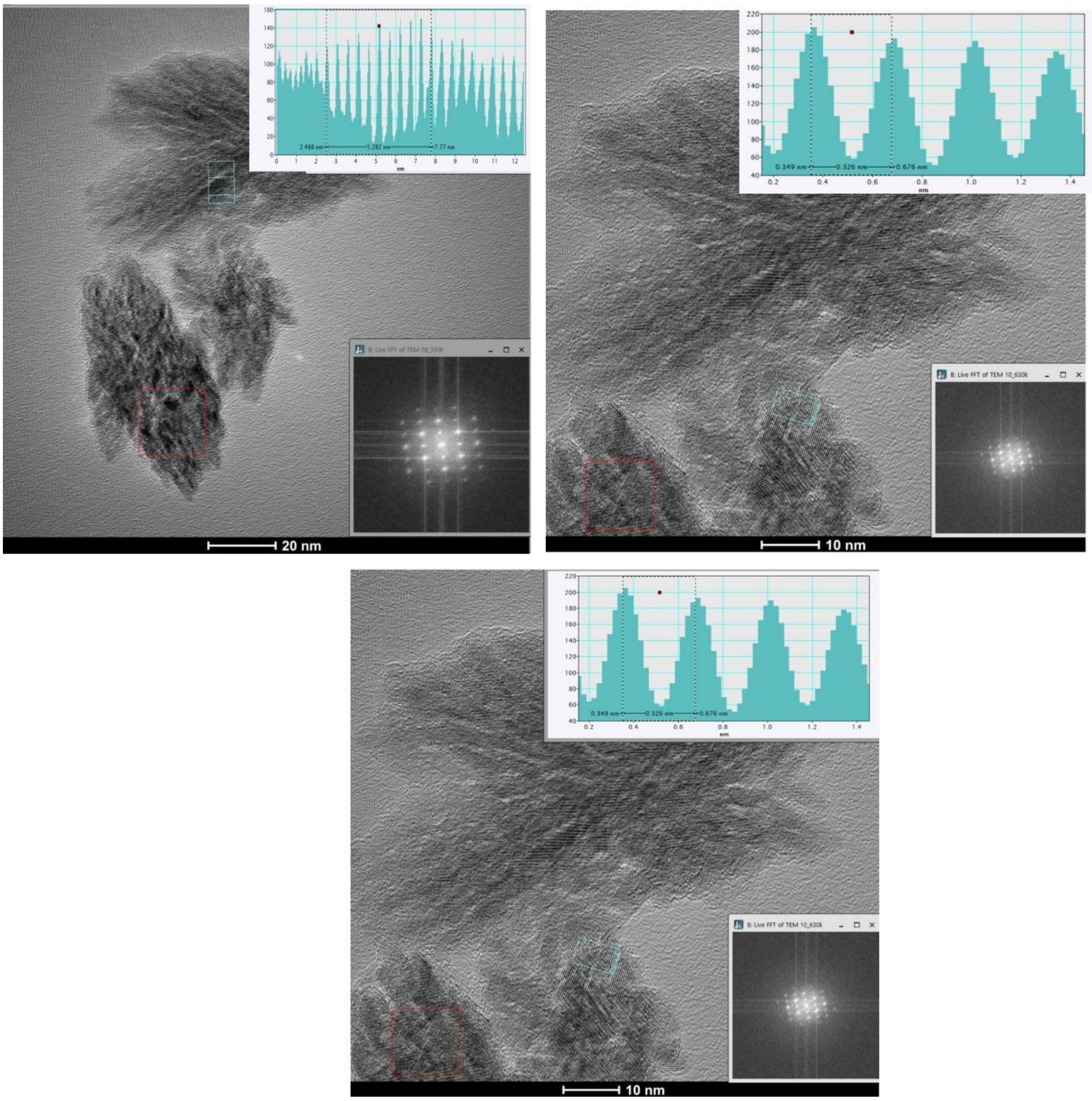

Figure S.I. AFM

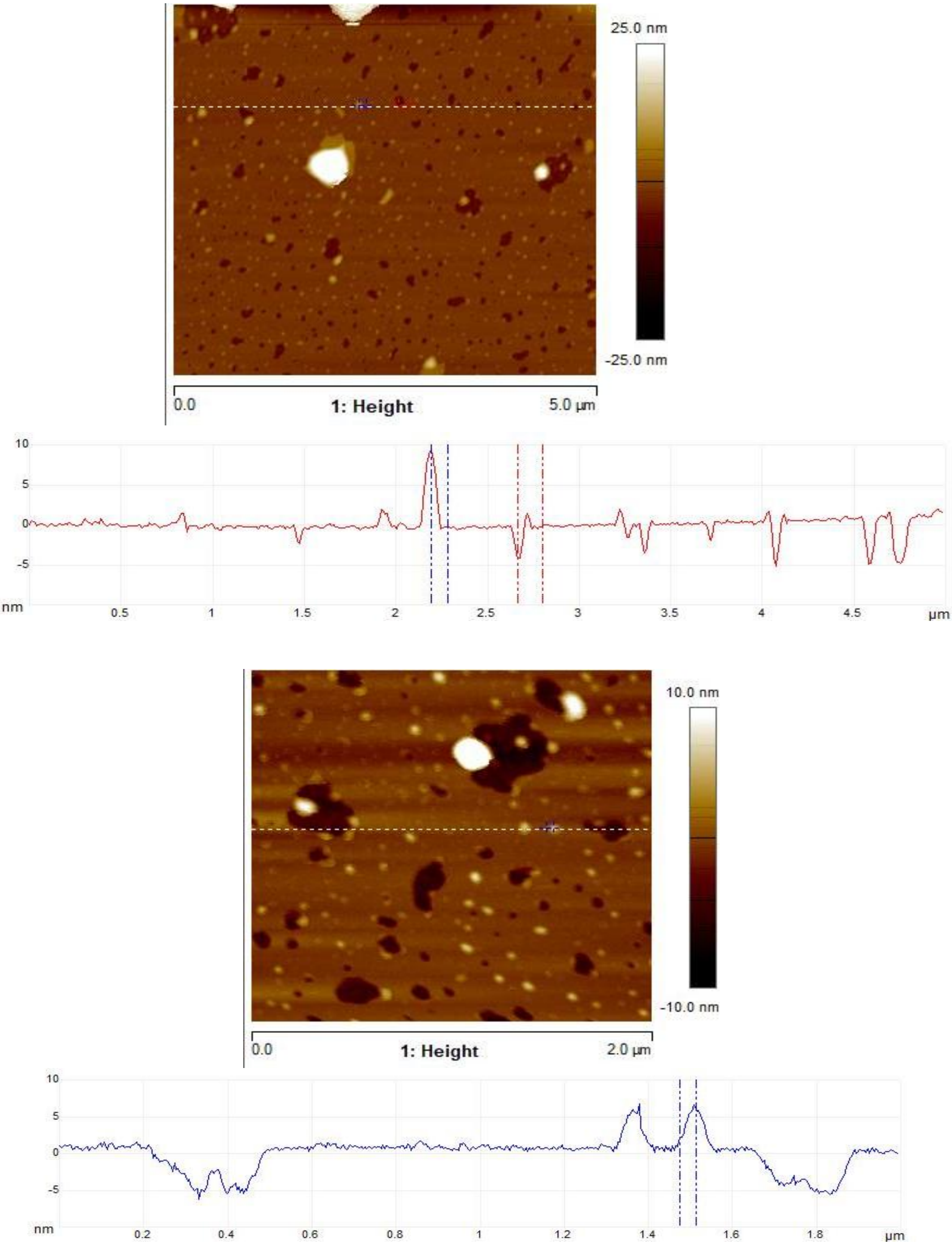

Figure SI Zr-O-HAADF Overlay

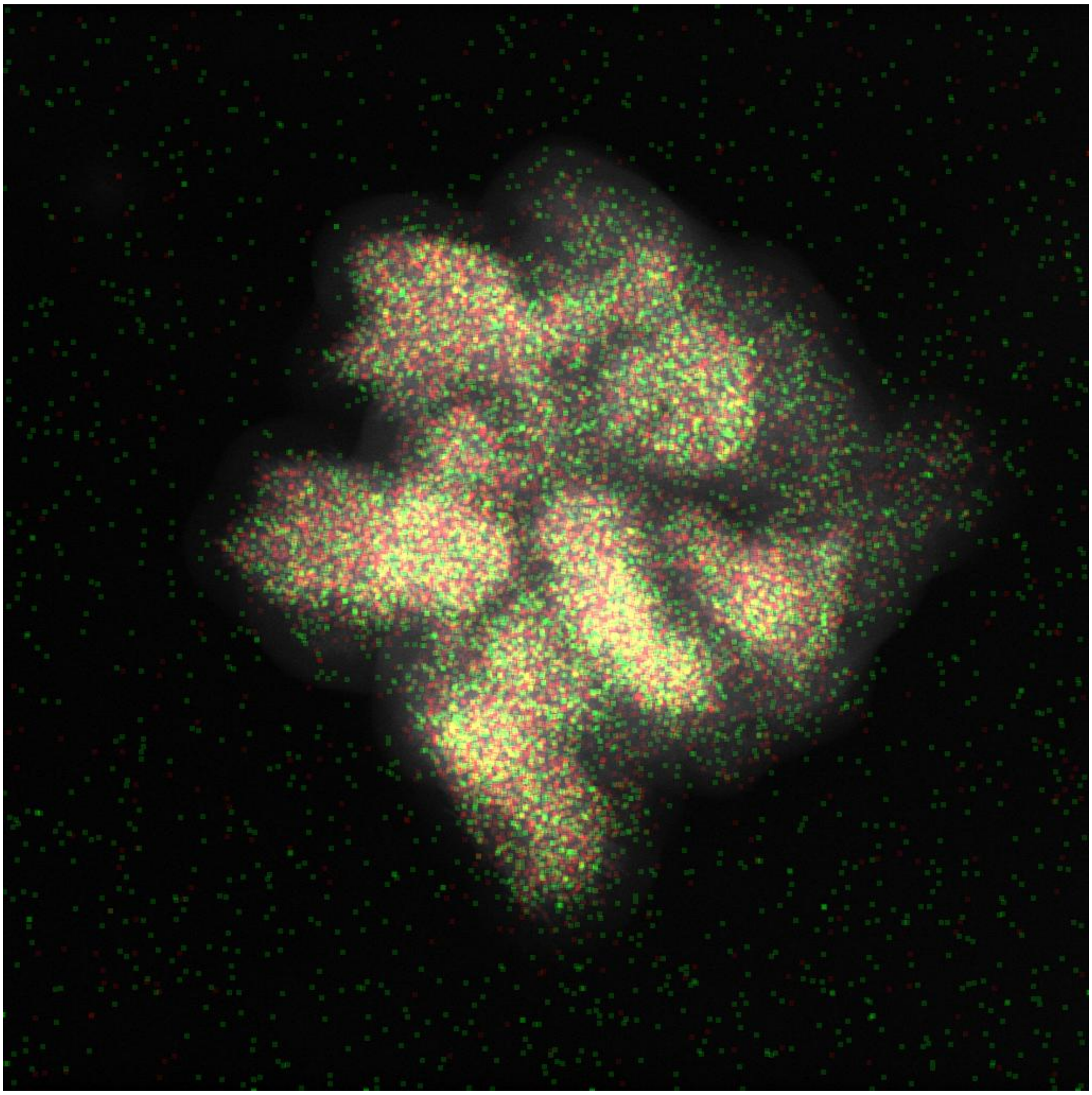

R: red, O: green

Figure S.I. DRX

Comparison of ZIPER DRX with that of PTCDA crystals

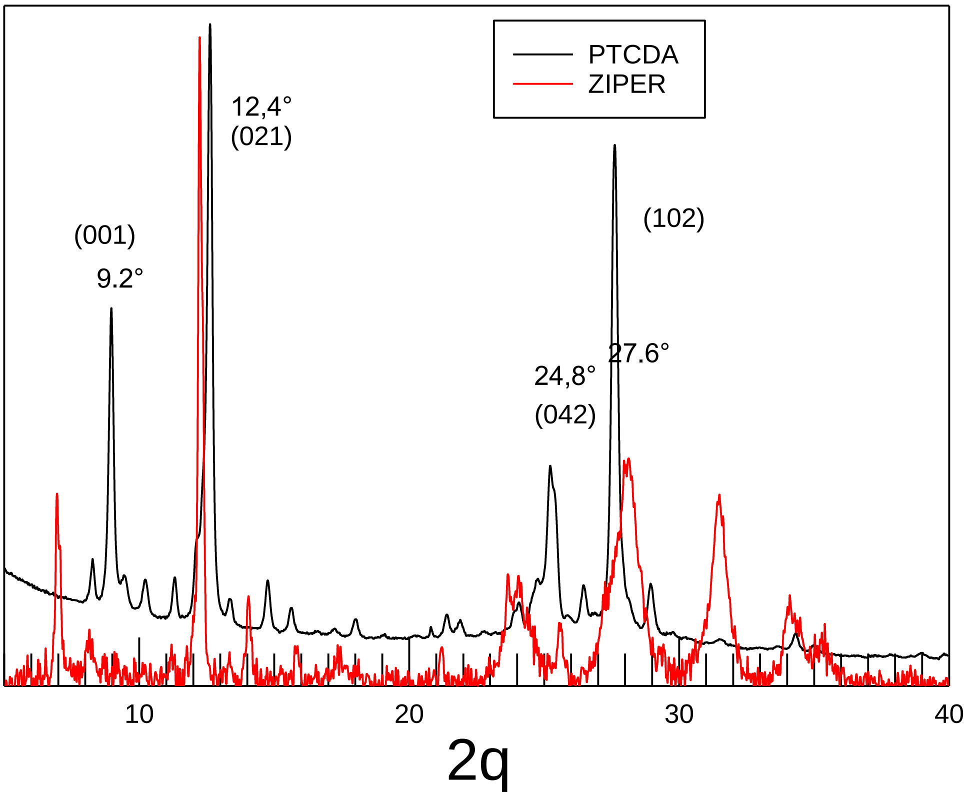

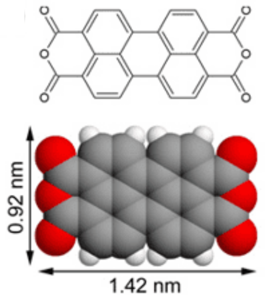

Figure S.I. FTIR

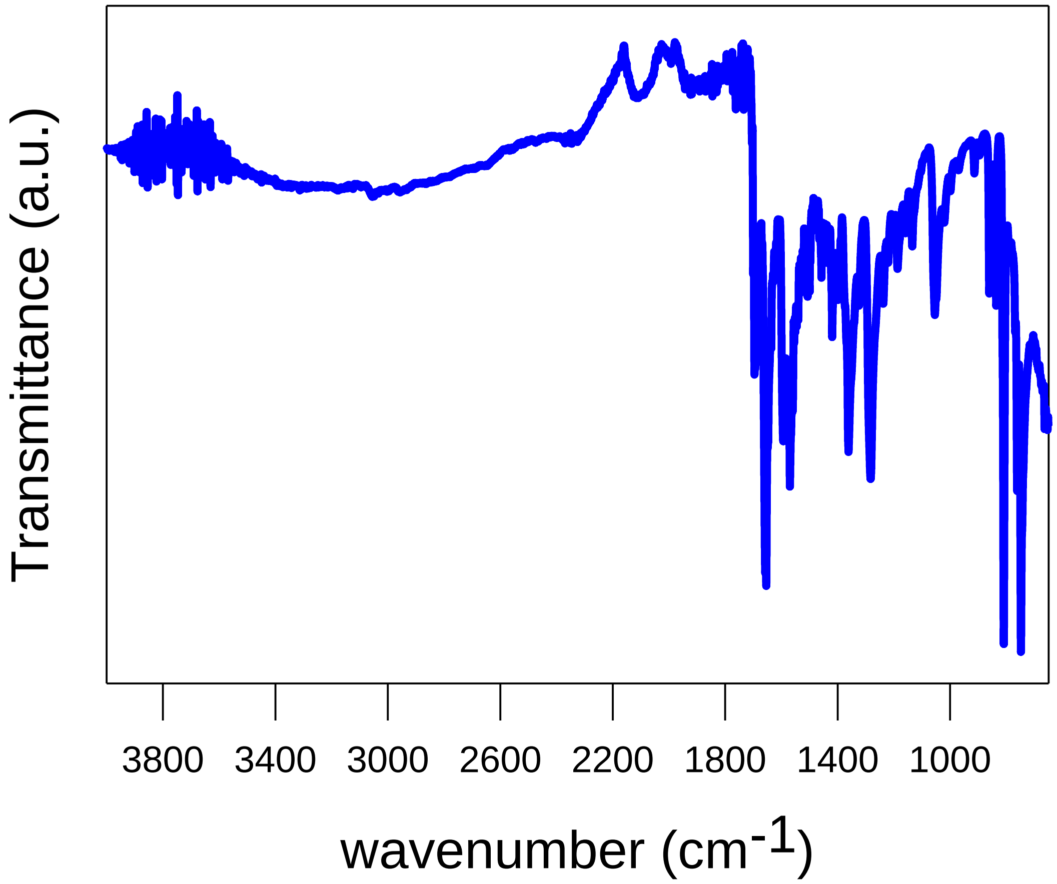

Figure S.I. XPS Survey

N 1s, O 1s, Zr 3d, Zr 4s, and C 1s XPS signals of ZIPER samples.

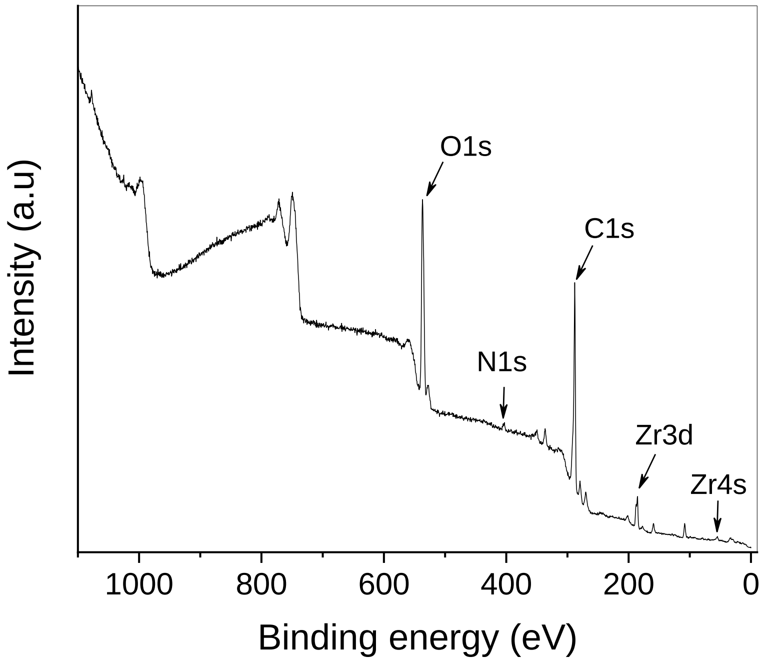

Figure S.I. EXAFS spectra of ZIPER and ZrO_2

Fourier Transformed of the EXAFS oscillation, measured at the Zr-K-edge for ZIPER (red line) and ZrO_2 monoclinic reference (green line).

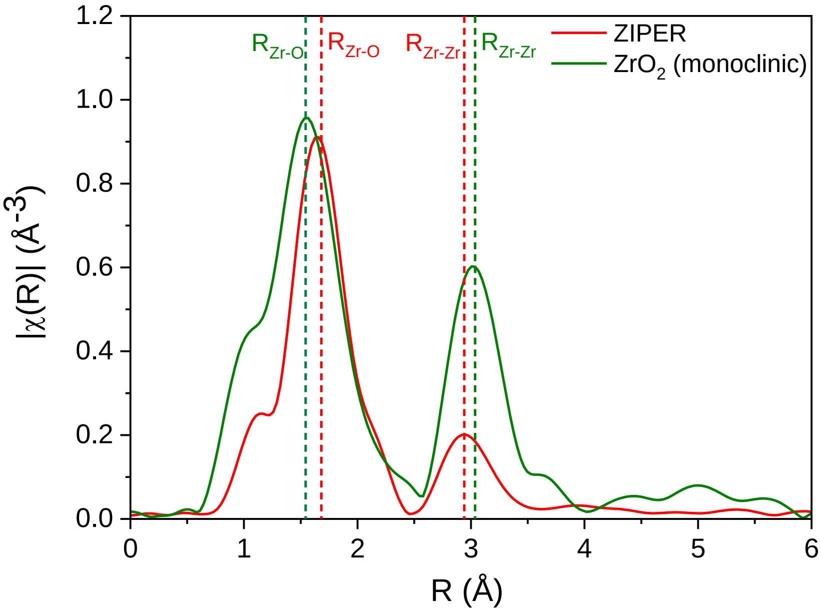

To further clarify the local structure of Zr in the material, we performed EXAFS measurements at the Zr K-edge, comparing the Fourier-transformed spectra of the Zipper sample with that of monoclinic ZrO_2 (as shown in the Figure S.I. EXAFS spectra of ZIPER and ZrO_2). The first-shell coordination (Zr–O) appears with comparable amplitude in both samples, though a slight shift in peak position is observed for the Zipper sample, suggesting differences in the local Zr–O bonding environment. More importantly, the second coordination shell, attributed to Zr–Zr scattering in crystalline ZrO_2 (coordination number ~12), appears significantly attenuated in the Zipper sample. EXAFS fitting yields a Zr–Zr coordination number of approximately 4, which is consistent with Zr_6O_8-type cluster environments and incompatible with extended ZrO_2 domains. This supports the presence of Zr in a structurally distinct, molecular-like coordination environment rather than in segregated zirconia nanoparticles.

Figure S.I. Sorption isotherms

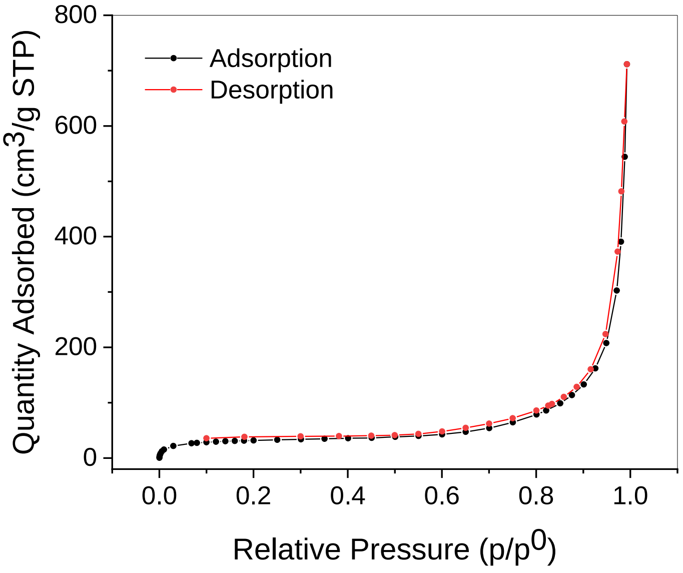

Figure S.I. Excitation and emission spectra

Emission spectra of ZIPER suspensions in DMSO at excitation wavelengths spanning from 450 to 750 nm (as denoted in the legend of the graph). Within the experimental error, the spectrum shows no variations with the excitation wavelength from 340 to 550 nm.

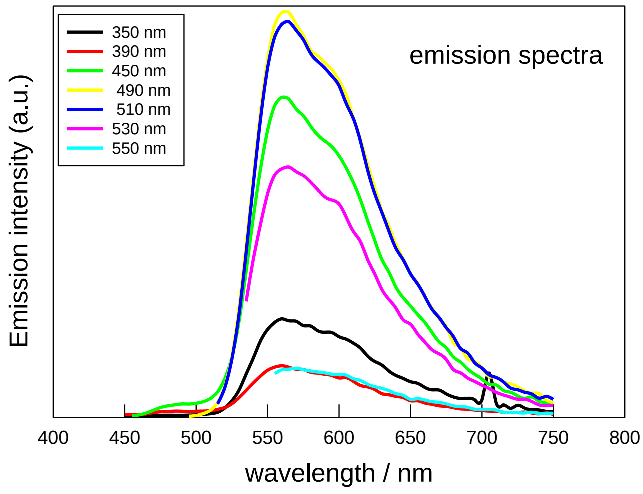

Excitation spectra of ZIPER suspensions in DMSO obtained upon detection at various emission wavelengths (as denoted in the legend of the graph). All spectra were recorded in suspensions of < 0.05 absorbance at 350 nm.

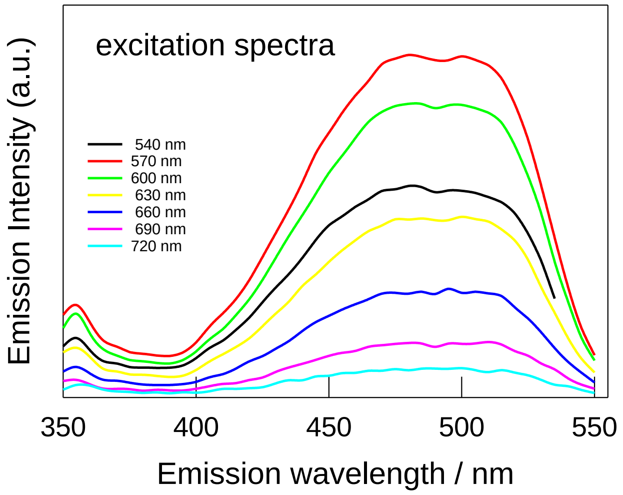

Figure S.I. Absorption spectrum

Comparison of the absorption and excitation spectra of ZIPER in DMSO suspensions.

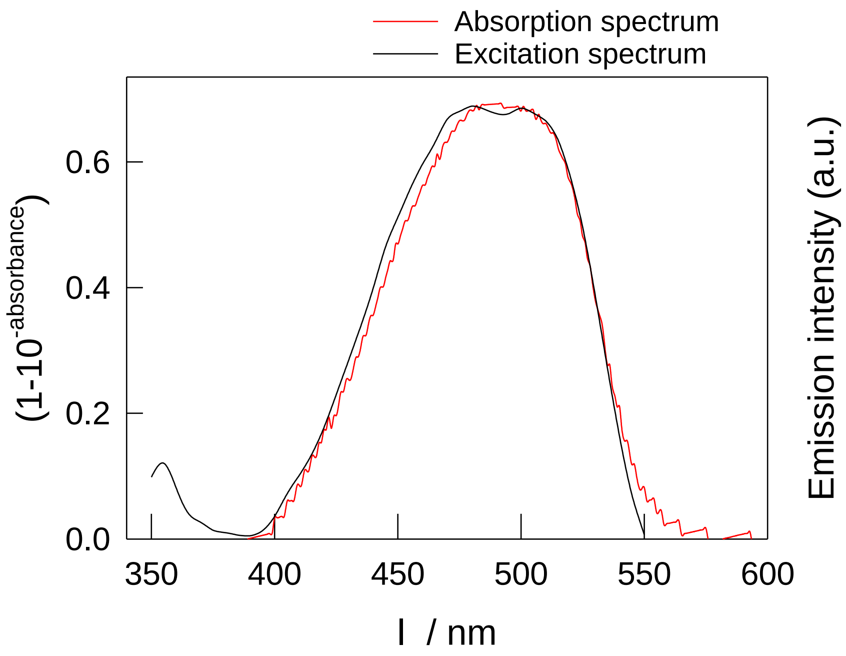

Excitation spectrum taken observing luminescence at 570 nm emission (black line) and absorption spectrum (red line) of ZIPER suspensions in DMSO.

Figure S.I. Effect of concentration on ZIPER PL

Normalized room temperature emission spectra of ZIPER suspensions in DMSO containing different amounts of nanoparticles in the range from 1.2 to 50 mg/L.

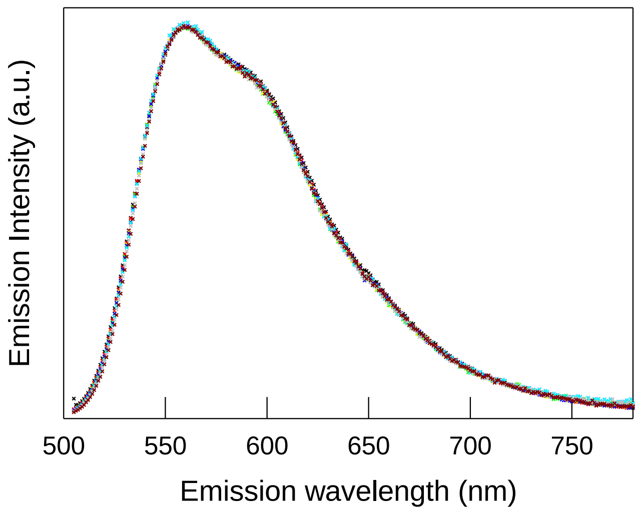

Figure S.I. Vibronic progressions

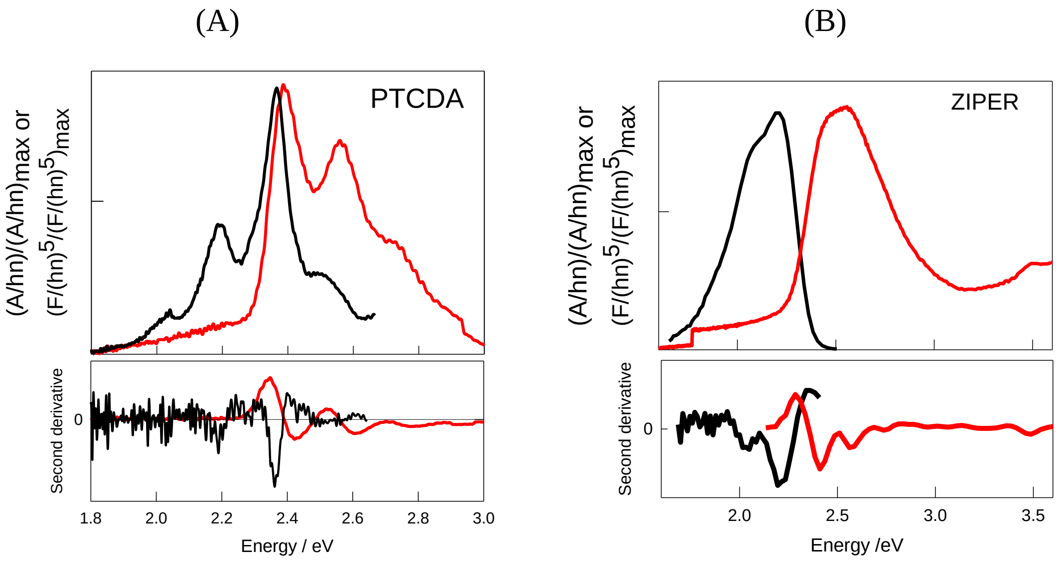

Top figures: Absorption (red lines) and photoluminescence (black lines) spectra (350 nm excitation light) of DMSO suspensions of diluted PTCDA suspensions at RT (A), and ZIPER suspensions at RT (B). *Bottom figures*: second derivative of the corresponding absorption and emission spectra.

Figure S.I. Tauc plot

The Tauc method is a simple and robust method to measure the energy gap from the absorption spectrum of nonmolecular semiconductors and organic molecular solids. However, it is suggested in the literature that, approaches based on the Tauc method to distinguish between direct and indirect gaps may not be accurate for MOF-type structures. Therefore, since the plot of $[2.3\times A\times h\upsilon]^2$ against the photon energy $h\upsilon$, with A the absorption at light energy $h\upsilon$, clearly showed a distinguishable linear part, the linear line extrapolation to the horizontal axis might be taken as the E_g estimation. Thus calculated E_g = 2.32 eV is in coincidence with the maximum wavelength of ZIPER absorption-emission superposition.

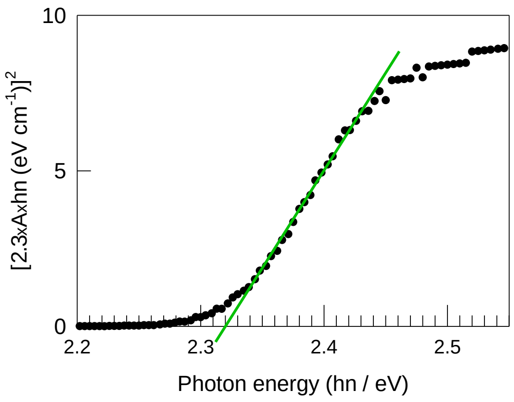

Tauc plot as obtained from ZIPER absorption spectrum.

Reductive Quenching

TEOA:

Stern-Volmer plots, eq. (1), of the ratio of the fluorescence decay in absence and presence of TEOA (τ_o/τ) *vs* [TEOA], yield a straight line (r^2=0.999) from which slope a Stern Volmer constant K_{SV}= (8.05 ± 0.1) M^{-1} is obtained (see Figure 5A). The higher quenching rates observed for plots of the relative PL intensity in the absence and presence of quencher (I_0/I) *vs* [TEOA] than those observed for dynamic quenching experiments (see Figure 5A), strongly support the formation of either non-emissive ground state complexes or the effect of adsorbed quencher molecules in some close distance to the excited probe, not forming a stable bimolecular complex but subjected to a very fast deactivation leading to a static quenching effect. This effect, known as the *sphere-of-action model* is described by eq. (2). Therefore, fitting of the steady state quenching data to eq. (2) (r^2=0.996) yields K_{SV} = (8.0 ± 0.6) M^{-1} in line with dynamic quenching experiments, and a sphere of action V_q = (2.8 ± 0.1) nm^3. From the latter value, an action distance d_q= 0.87 nm is estimated, which is of the order of the length of a perylene aromatic nuclei (1.4×0.42 nm^2).[48]

TEA:

For TEA, Stern-Volmer plots obtained from steady state quenching experiments depict a downward/negative deviation, as shown in Figure 5B *inset*. Negative deviations may be associated with emitters which cannot be reached by quencher molecules. The PL intensity I in the presence of quencher is given by the Lehrer equation, equation (3), provided that no static quenching is present.[49] A plot of the Lehrer equation for the data obtained for TEA quenching of ZIPER's PL, see Figure 5B, could be well fitted to eq. (3) (r^2 = 0.986) considering a Stern-Volmer constant K_{SV} = (12 ± 2) M^{-1} and a fraction f = 0.91 ± 0.06 of accessible chromophores for quenching. It should be noted that, in cases when chromophores cannot be accessed by the quencher, fluorescence decay in the

presence of added quencher is bi-exponential with two fluorescence decay components, $\tau_1 = \tau_0$ and $\tau_2 = \tau_0 \times (1+K_{SV}[Q])^{-1} < \tau_0$ with amplitudes proportional to $1-f$ and f, respectively.[47] Due to the experimentally accessible narrow decay range and the low proportion of non-quenchable chromophores, such biexponential analysis was possible mainly for the higher quencher concentrations. Stern-Volmer plots of thus obtained τ_2 values, see Figure 5B *inset*, depict straight lines (r^2=0.999) with K_{SV} = (14.8± 0.3) M^{-1} in line with that obtained from steady state experiments.

Interestingly, meanwhile TEOA can quench all ZIPER's chromophores, TEA cannot access *ca.* 9% of the emitters. It is reported in the literature that diffusion-controlled luminescence quenching of a $Ru(bpy)_3^{2+}$-derived bridging ligand MOF using a series of amines of different sizes as quenchers,[51] showed that the largest amines were found to be too large to enter the MOF channels. Since TEOA is expected to be larger than TEA because of OH terminal groups, quencher size seems not to be the main reason of our observations. However, considering the strong difference in the dielectric constants at 25 °C for TEA and TEOA, ε = 2.42 and 37.7, respectively, it may be expected that *ca.* 9% of the chromophores are in hydrophilic zones (pores) disturbing emitters interaction with TEA.

DPA:

Quenching of ZIPER PL by DPA is observed in continuous irradiation experiments, see SI Figure Steady State Quenching. Fitting of the plots of (Io/I) vs [DPA] to the Stern Volmer eq. (1) (r^2=0.94) yields KSV = (35 ± 3) M^{-1}. Depletion of DPA was also effectively demonstrated in the logarithmic plot of SI Figure Steady State Quenching *inset*.

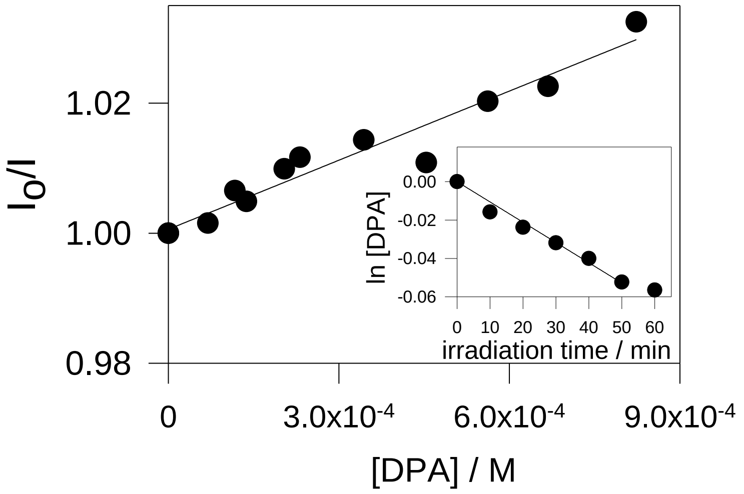

SI Figure Steady State Quenching: Quenching experiments by DPA (●) Plot of the ratio between the PL intensity of ZIPER in the absence and presence of DPA (I_0/I) *vs* [DPA] and fitting to the Stern Volmer equation (r^2=0.94). *Inset*: Plots of the natural logarithm of DPA concentration in experiments with ZIPER.

Dynamic quenching is not observed, see SI Figure Dynamic Quenching, though, due to DPA solubility limitations, K_{SV} constants of the order of 30 M^{-1}, and the fast decay of ZIPER's PL, decays in the presence and absence of DPA are within the error of our equipment.

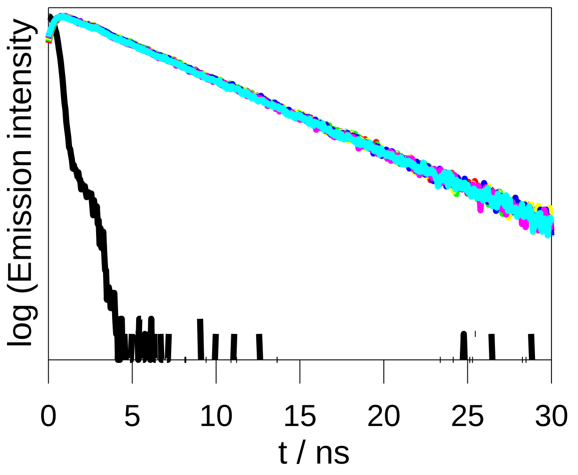

SI Figure Dynamic Quenching: PL decay lifetimes in the presence and absence of DPA

Methyl Viologen quenching.

Steady state results were also analyzed considering that not all the emitting chromophores are reachable by MV^{2+}. Fitting to the Leher eq. (3) (r^2= 0.987) yielded K_{SV} = (76 ± 30) M^{-1} and a fraction f = 0.3 ± 0.1 of accessible chromophores for MV^{2+} quenching. However, an analysis of the fluorescence decay to a bi-exponential function with two decay components, $\tau_1 = \tau_0$ and $\tau_2 < \tau_0$ with amplitudes proportional to $1-f$ and f, respectively,[1] and further fitting of thus obtained τ_2 values to the Stern Volmer equation, did not return a K_{SV} in reasonable agreement with that of the steady state data. Therefore, the occurrence of both static quenching due to the sphere of action and the fact that not all ZIPER chromophores are accessible for MV^{2+} quenching cannot be discarded.

Hydrogen peroxide determination

Experiments were performed irradiating with green light (520 nm) ZIPER suspensions in DMSO of 0.3 absorbance at the excitation wavelength, containing 10 mM TEA or TEOA. After 1 h irradiation under air equilibrate conditions, 400 μl of the irradiated suspension was added to 1.8 ml Colestat™ solution and the mixture was incubated for 5 min at 37 °C. A ZIPER suspension prepare in identical conditions was exposed to 1 hr irradiation after a thoroughly bubbling with argon for 20 min, in order to analyze the effect of the confine O_2 within the metal-organic assemblies structure. Finally, the visible spectra were measured (see following figure) and the absorbance at 505 nm was taken for the estimation of H_2O_2 concentration. A solution consisting of 1.8 ml Colestat™ and 400 ul ZIPER suspension without irradiation was taken as a blank. To prove the involvement of the amines in the photocatalytic process for the generation of H_2O_2, ZIPER suspensions without the addition of TEA or TEOA were used as negative control under the same irradiation conditions. The following figure shows Colestat absorption spectrum as obtained after irradiation of ZIPER suspensions under the different conditions described. The error in the measure of H_2O_2 is 10 mM h^{-1}.

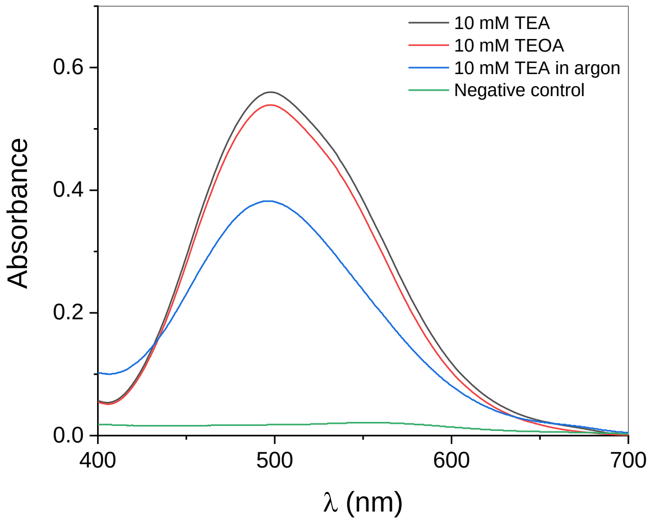

Detection of $MV^{\cdot+}$ after photoinduced electron transfer.

Because methyl viologen one-electron reduced species ($MV^{\bullet+}$) is stable for many hours in the absence of molecular oxygen and is easily detectable by visible absorption spectrometry[2] (extinction coefficient ε ~1.37×10^4 $M^{-1}cm^{-1}$ at 606 nm),[3] MV^{2+} is recognized as a versatile redox indicator in photoinduced electron transfer studies. Therefore, further experiments involving ZIPER PET in the presence of TEA or TEOA, two well-known sacrificial agents,[6,7] and MV^{2+} were performed. Accordingly, considering our previous findings (see main manuscript), 0.05 M of TEA (or TEOA) and 5×10^{-5} M MV^{2+} can quench almost 6.5% and < 0.4% of ZIPER PL, leading to a photoredox cycle described in Scheme S.I.1. Under these conditions, any formation of $MV^{\bullet+}$ is due to the reduction of MV^{2+} by the radical anion $ZIPER^{\bullet-}$, as shown in reactions (R5) in the redox cycle.

Scheme S.I.1.

Proposed redox cycle for PET experiments with [TEA], [TEOA] ~ 1000×[MV^{2+}], see text.

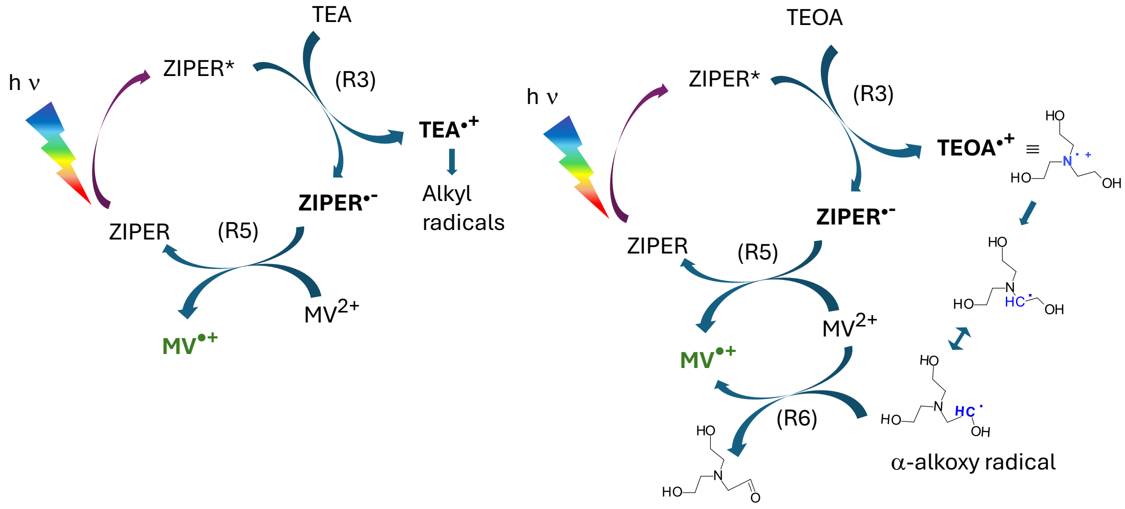

In fact, 500 nm light irradiation experiments of a ZIPER DMSO suspension of 0.1 absorbance at 500 nm, in the presence of 5×10^{-5} M MV^{2+} and 0.05 M TEA did not show the formation of $MV^{\bullet+}$. Contrary to these results, experiments under identical conditions but in the presence of TEOA show formation of $MV^{\bullet+}$ radical.[4,5]

The following Figure shows the increase of $MV^{\bullet+}$ concentration during the first 20 minutes continuous irradiation. The unexpected result might be understood considering TEA and TEOA degradation pathways. In Scheme S.I.1, both, $TEA^{\bullet+}$ and $TEOA^{\bullet+}$ radical cations are formed from the PET reaction with excited ZIPER*. The fast decomposition of radical cations from simple aliphatic amines might partially prevent reaction (R3) counter back-electron transfer. However, this process is highly dependent on the photosensitizer and the capability of aminyl radicals to rapidly decompose to alkyl radicals.[8,9] Therefore, experiments with TEA strongly suggest that $ZIPER^{\bullet-}$ is not available to reduce MV^{2+}. According to our previous discussions, two main reasons are responsible for this situation: the limited access of MV^{2+} to $ZIPER^{\bullet-}$ sites and the remaining concentration of O_2 inside the pores despite exhausting degasification.

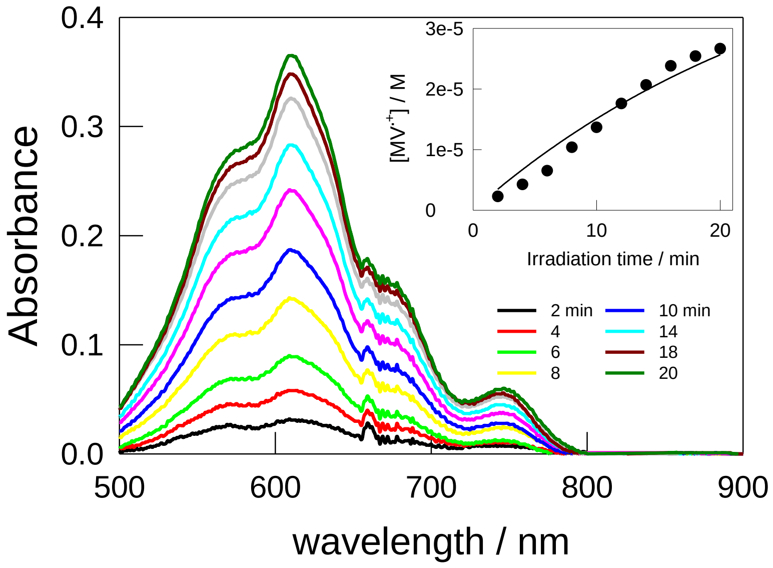

Figure. $MV^{\bullet+}$ formation in 500 nm light irradiation experiments of a ZIPER DMSO suspension of 0.1 absorbance at 500 nm, in the presence of 5×10^{-5} M MV^{2+} and 0.05 M TEOA.

On the other hand, it is well reported in the literature that, deprotonation of $TEOA^{\bullet+}$ leads to a fast rearrangement into a carbon centered α-alkoxy radical displaying a significant reductive power of *ca.* - 1V. An extremely simplified mechanism for $MV^{\bullet-}$ formation in the presence of TEOA assumes a fast $TEOA^{\bullet+}$ rearrangement into carbon centered α-alkoxy radicals which can further reduce MV^{2+} outside ZIPER´s pores, reaction (R6) in Scheme S.I.1. Since α-alkoxy radicals are able to scavenge O_2 with diffusion-controlled rates, they protect $MV^{\bullet+}$ radical anions from being oxidized back to MV^{2+} by O_2. In fact, corresponding experiments with [TEOA] ~ 0.0005 M do not show $MV^{\bullet+}$ formation.
